# Methylome decoding of RdDM-mediated reprogramming effects in the Arabidopsis MSH1 system

**DOI:** 10.1186/s13059-022-02731-w

**Published:** 2022-08-04

**Authors:** Hardik Kundariya, Robersy Sanchez, Xiaodong Yang, Alenka Hafner, Sally A. Mackenzie

**Affiliations:** 1 Department of Biology, The Pennsylvania State University, 362 Frear N Bldg, University Park, PA 16802 USA; 2 School of Horticulture and Plant Protection, Yangzhou University, Yangzhou, Jiangsu China; 3 Plant Biology Graduate Program, The Pennsylvania State University, University Park, PA USA; 4 Department of Plant Science, The Pennsylvania State University, University Park, PA USA

**Keywords:** DNA methylation, Epigenetic, sRNA, Vigor, Stress response, Phenotypic plasticity

## Abstract

**Background:**

Plants undergo programmed chromatin changes in response to environment, influencing heritable phenotypic plasticity. The RNA-directed DNA methylation (RdDM) pathway is an essential component of this reprogramming process. The relationship of epigenomic changes to gene networks on a genome-wide basis has been elusive, particularly for intragenic DNA methylation repatterning.

**Results:**

Epigenomic reprogramming is tractable to detailed study and cross-species modeling in the *MSH1* system, where perturbation of the plant-specific gene *MSH1* triggers at least four distinct nongenetic states to impact plant stress response and growth vigor. Within this system, we have defined RdDM target loci toward decoding phenotype-relevant methylome data. We analyze intragenic methylome repatterning associated with phenotype transitions, identifying state-specific cytosine methylation changes in pivotal growth-versus-stress, chromatin remodeling, and RNA spliceosome gene networks that encompass 871 genes. Over 77% of these genes, and 81% of their central network hubs, are functionally confirmed as RdDM targets based on analysis of mutant datasets and sRNA cluster associations. These *dcl2/dcl3/dcl4*-sensitive gene methylation sites, many present as singular cytosines, reside within identifiable sequence motifs. These data reflect intragenic methylation repatterning that is targeted and amenable to prediction.

**Conclusions:**

A prevailing assumption that biologically relevant DNA methylation variation occurs predominantly in density-defined differentially methylated regions overlooks behavioral features of intragenic, single-site cytosine methylation variation. RdDM-dependent methylation changes within identifiable sequence motifs reveal gene hubs within networks discriminating stress response and growth vigor epigenetic phenotypes. This study uncovers components of a methylome “code” for de novo intragenic methylation repatterning during plant phenotype transitions.

**Supplementary Information:**

The online version contains supplementary material available at 10.1186/s13059-022-02731-w.

## Background

Plants deploy a range of seed dispersal mechanisms that can span significant distance and environmental variation [1], necessitating robust environmental adaptation strategies. Successful germination and seedling establishment in a new location favors inducible and sustainable resilience mechanisms to facilitate survival under diverse environmental conditions. Modeling heritable phenotypic response assumes a plant’s ability to manage internal metabolic stochasticity in a manner that provides for phenotype stability (canalization) together with the requisite plasticity to respond to environmental change [2, 3]. It is thought that plants persist in new and changing environments through epigenetic processes [4, 5], but directional targeting of specific gene networks for enhanced phenotypic resilience in plants has not been previously defined.

Targeted epigenetic changes in plants take place, in part, through the RNA-directed DNA methylation (RdDM) pathway. This process effects de novo DNA methylation within transposable element and heterochromatic regions of the genome [6, 7], with less clear influence on genic methylation. Small RNAs (sRNA) associated with the RdDM pathway establish target sites and are generated by the activity of *DCL3* in the canonical pathway [6], and *DCL2* and *DCL4* in associated pathways [7]. Site-specific DNA methylation is directed by the methyltransferase DRM2 [8] in a process that links to histone modifications influencing local chromatin architecture. These effects involve the histone deacetylase HDA6 [9]. HDA6 is a component of chromatin-mediated stress response [10, 11], presumably participating in re-establishment of chromatin homeostasis following environmental or developmental changes [12].

**Fig. 1:**
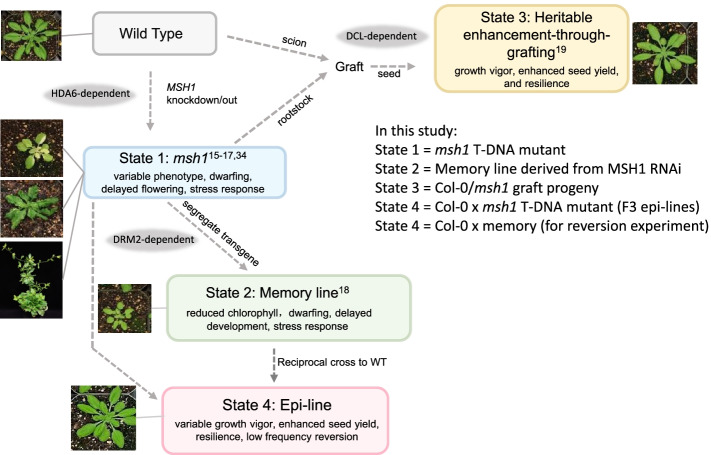
The *msh1* system is composed of four *msh1*-derived epigenetic states. In Arabidopsis, four distinct plant states originate from *MSH1* knockdown or knockout. States 1 and 2 derive directly from *msh1* disruption, resulting in highly stress-responsive phenotypes. State 1 at short daylength is variable, including a low-frequency “perennial-like” phenotype [16]. States 3 and 4 involve interaction of *msh1*-modified and naïve (wild type) genomes through grafting or crossing, resulting in growth vigor phenotypes. Genetic evidence of RdDM dependence is indicated at relevant transitions in gray shading

*MSH1* is a plant-specific, nuclear-encoded gene for a DNA-binding protein [13] that maintains mitochondrial [14] and plastid [15] genome stability. Disruption of the plastid-targeted MSH1 protein causes plant developmental and epigenetic reprogramming [16, 17] and a variable, stress-responsive, daylength-sensitive growth phenotype (state 1) (Fig. 1). The *msh1* reprogramming process is *HDA6*-dependent, such that an *msh1,hda6* double mutant is lethal under standard (12-h daylength) growth conditions [18]. RNAi knockdown of *MSH1* and subsequent transgene segregation leads to recurrently heritable, nongenetic *msh1* “memory” (state 2). The *msh1* memory state is a *DRM2*-dependent condition of reduced growth, delayed maturity, and chronic abiotic stress response [18].

Grafting an isogenic wild type scion to *msh1* rootstock produces progeny with measurably enhanced growth vigor, seed production, and resilience (state 3). This phenotype is markedly different from *msh1*(state 1) and derived memory (state 2). The graft progeny growth vigor, referred to as heritable enhancement through grafting (HEG), is sustained over multiple successive generations [17]. HEG effects depend on transgenerational sRNA transmission. Progeny growth and differential methylation outcomes are obviated by including *dcl2/dcl3/dcl4* triple mutations with *msh1* in the rootstock [19]. A fourth *msh1*-derived nongenetic state exists, detailed in this report. This state arises from crossing of *msh1* to isogenic wild type, leading to a range of enhanced growth vigor in the epi-line progeny. In epi-lines, these effects are more variable and diminish after 5 to 6 generations, with evidence of sporadic reversion to a condition resembling state 2 memory as shown in this study.

Here, we exploit the MSH1 system as a model to address two fundamental questions of plant phenotypic plasticity: What are the criteria for identifying plant de novo RdDM gene targets that influence directional phenotype adjustment, and do these epigenomic effects lend themselves to prediction? We integrated nongenetic plant phenotype variation, DNA methylation, sRNA, mutant, and gene expression datasets from the four *msh1*-derived nongenetic states, to home in on putative target pathways that participate in *msh1* developmental reprogramming. With a methylation analysis procedure enhanced for sensitivity, we discriminated gene networks undergoing directional changes in methylation and transcription. These networks appeared to underpin plant phenotype transitions. The DNA methylation analysis incorporated signal detection, machine learning, and sRNA and RdDM mutant datasets to classify candidate RdDM target loci that participate in *msh1* effects without regard to cytosine context or numbers of proximal methylation changes at a site. High-resolution analysis of these putative targets pinpointed differential methylation positions within identifiable sequence motifs to discriminate the four *msh1*-derived states. These observations support a model of *msh1*-triggered epigenetic effects as targeted, programmed actions that may serve to promote phenotype plasticity in a manner far more precise than has been previously reported.

## Results

### *msh1* epi-lines comprise a distinct nongenetic state based on phenotype

**Fig 2:**
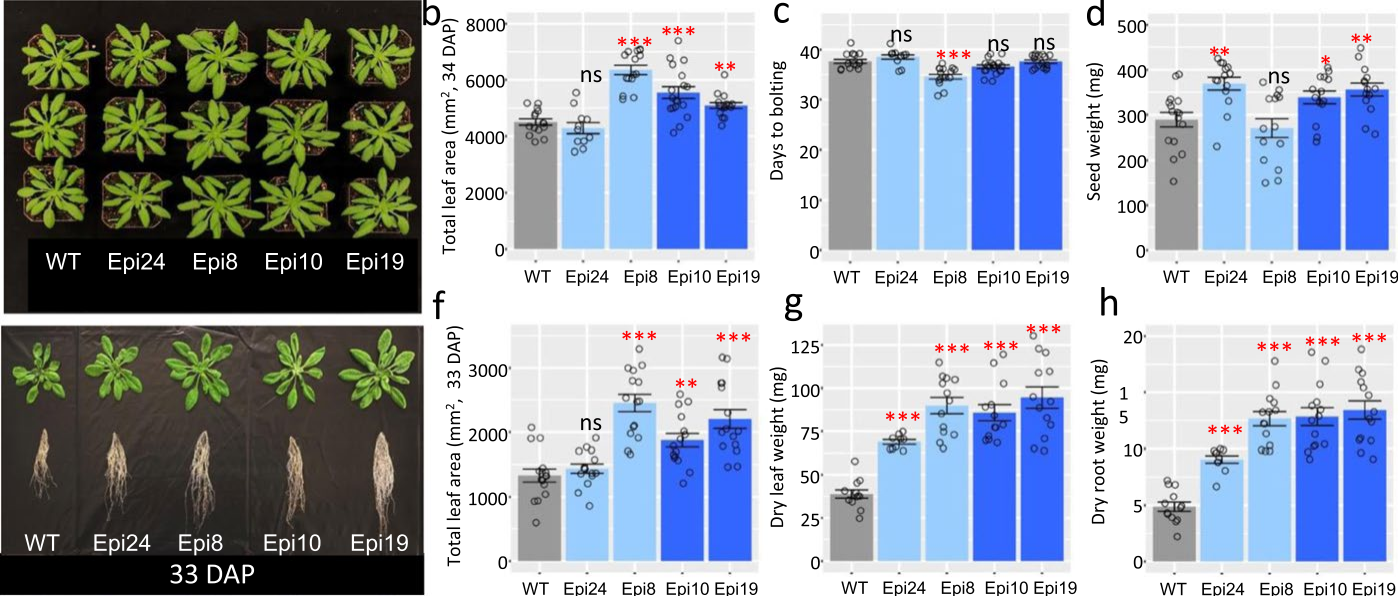
Characteristics of epi-lines derived by crossing *msh1* T-DNA mutant with isogenic wild type. **a** The phenotypes of different epi-line F3 populations at 34 DAP. The lines derive from WT x *msh1* crosses, with Epi 24 and Epi 8 from one parental cross, and Epi 10 and Epi 19 from a second parental cross. **b** Total leaf area (34 DAP), **c**, days to bolting, and **d**, seed weight (mg) are shown for the four populations along with WT control. **b–d** Bars represent means ± SE. The Mann–Whitney *U*-test with two-sided alternative hypothesis was used to test significance of the difference of mean between each Epi F3 population and WT control. **e** Root phenotype of the four different Epi F3 populations grown in sand (33 DAP). **f** Total leaf area (33 DAP), dry leaf weight (mg), and dry root weight (mg) are shown for the four populations grown on sand along with WT control. **f–h** Bars represent means ± SE. The Mann–Whitney *U*-test with two-sided alternative hypothesis was used to test significance of the difference of mean between each Epi F3 population and WT control. Significance codes: *p < 0.05, **p < 0.01, ***p < 0.001, ns – not significant

Manipulation of the *msh1* mutant leads to four distinct phenotypes, with states 1 and 2 characterized by slowed growth, delayed flowering, and persistent stress response, and states 3 and 4 producing enhanced growth vigor and greater seed set over wild type (WT) (Fig. 1). State 4 results from direct or reciprocal crossing of Col-0 *msh1* mutant (state 1) or memory line (state 2) x Col-0 WT and generation of epi-F2 and epi-F3 families in Arabidopsis. Similar results were obtained regardless of the direction of the cross, which indicates that these progeny phenotype changes do not derive from altered plastid genome effects. Regardless, we used Col-0 WT as female in crosses presented in this study to retain wild type cytoplasm. Progeny populations showed more variable distribution of growth-enhanced phenotypes within the F_2_ generation than occurs in state 3 graft progeny [19], and individual epi-line populations could be categorized with either enhanced vegetative growth, greater seed set, or both (Fig. 2; Additional file 1: Fig. S1).

We followed 4 F3 populations of cross-derived epi-lines. Epi 8 and Epi 24 were sibling lines from one WT x *msh1* cross event, and Epi 10 and Epi 19 were sibling lines from a second WT x *msh1* cross. All four F3 epi-lines showed uniform phenotypes within each population, but significant variation between the four populations (Fig. 2; Additional file 1: Fig. S1). Epi-line populations were increased in aboveground vegetative growth and underground root development (Fig. 2a, e). Three of the populations, Epi 8, 10, and 19, had significantly higher total leaf area than the WT control (Fig. 2b, f), and all four populations had higher dry leaf weight than WT (Fig. 2g). The four populations also showed higher dry root weight than WT (Fig. 2h).

Enhanced reproductive growth occurred in the three epi-lines, Epi 8, 10, and 19, while Epi 8 showed early flowering (Fig. 2c). Importantly, Epi 8 and Epi 24, full-sib populations from the same original cross, showed distinct phenotypes. Epi 24 was highest in seed weight and lowest in leaf area among the epi-lines, while Epi 8 was highest in leaf area and lowest in seed weight. These observations suggest that nongenetic growth vigor can vary in vegetative versus reproductive allocations within closely related populations, and we focused on Epi 8 versus Epi 24 for more detailed studies.

**Fig. 3:**
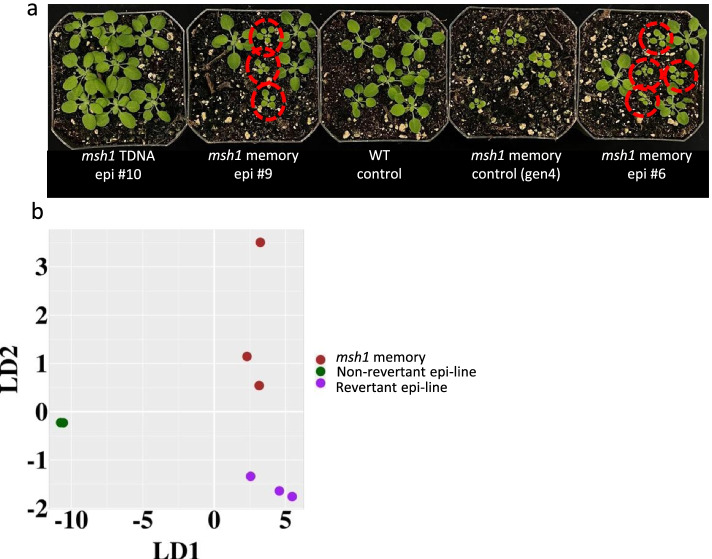
Reversion phenotype in Arabidopsis. **a** Plant growth phenotype of three F3 epi-line populations, Epi 10 derived by crossing to *msh1* T-DNA mutant, and Epi 6 and Epi 9 derived by crossing to *msh1* memory line. Dashed circles indicate putative revertants. Col-0 wild type and *msh1* memory are shown as controls. **b** Principal component analysis-linear discriminant analysis of methylome data from three epi-line (non-revertant) and three revertant full-sib F3 (state 4) progeny compared to three independently derived *msh1* memory (state 2) plants

The epi-line phenotype receded back to wild type by the fifth or sixth (S5, S6) generation. On occasion, we observed sporadic, low-frequency incidence of reversion to a condition resembling memory (state 2) phenotype (Fig. 3a). Putative revertants displayed reduced growth rate, altered leaf morphology, and enhanced stress response. Frequency of these putative reversion events ranged from 15 to 19% in Arabidopsis (Table 1). We have not observed this type of reversion phenomenon in graft progeny to date, with all graft progeny outcomes resulting in either enhanced growth or wild type growth phenotypes [17, 19].

**Table 1:** Reversion frequencies within epi-lines

Lines tested*	Population	Reversion frequency
Epi 9 (memory)	epi-F3	20/106 (18.87%)
Epi 6 (memory)	epi-F3	17/111 (15.32%)
Epi 10 (T-DNA)	epi-F3	0/77
*msh1* memory	Gen 4	35/35 (control)
WT Col-0		0/55 (control)

* In Lines tested, T-DNA refers to *msh1* T-DNA insertion mutant used in cross as control

The reversion phenomenon implies that the epi-line state 4 and memory state 2 conditions are closely related. Reversion occurred in only epi-line populations deriving from crosses to an *msh1* memory line (state 2) pollen parent, but not with *msh1* null mutant (state 1) as pollen parent (Table 1). We interpret this observation to indicate that *msh1* signal is weaker in the memory line than null mutant, leading to a less stable epi-line outcome. This interpretation would be consistent with the relatively weaker methylome repatterning effects reported in *msh1* memory (state 2) relative to *msh1* (state 1) [18].

### The *msh1* states 1 to 4 comprise discrete epigenetic phases by whole-genome methylome analysis

Significant changes in DNA methylation were detected in the four Arabidopsis epi-lines (F3), with gene-associated changes predominantly in CG context (Additional file 1: Fig. S2) and variable hyper and hypomethylation in CHH context within and between epi-lines (Additional file 1: Fig. S3). To estimate the relationship of intragenic methylation changes with plant phenotype, we used high-resolution methylome analysis. The procedure incorporates signal detection and machine learning to discriminate high-probability, treatment-associated methylation changes within gene regions. We consider three features of this analysis to be vital to this study: (1) The analysis focused on intragenic methylation repatterning without exclusion of single cytosine methylation changes. (2) DNA methylation variation within wild type samples was measured and subtracted to allow discrimination of treatment-associated (*msh1*-derived) methylation changes; and (3) individual cytosine methylation status, reflecting the outcome of pooled cell types, was analyzed as probability distributions using Hellinger divergence [20] rather than binary classification. The resulting datasets of treatment-associated differentially methylated positions (DMPs) could then be used for unbiased comparative analysis in all subsequent steps.

**Fig. 4:**
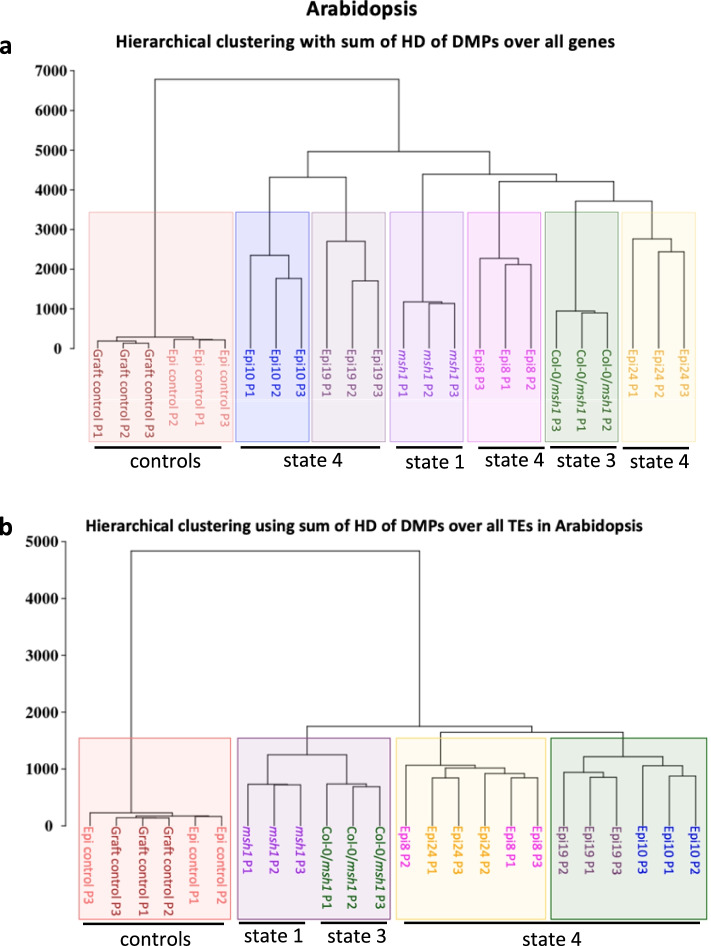
Discrimination of methylation repatterning among different *msh1* states. **a** Hierarchical clustering results with genic methylome data from three different *msh1* states in Arabidopsis: *msh1* null mutant (state 1), Col-0/Col-0_*msh1*_ graft progeny (state 3), Epi F3 populations (state 4), and relevant Col-0 controls. **b** Hierarchical clustering results with TE DMP data from the same Arabidopsis datasets presented in panel **a**. Individuals were represented as vectors of the sum of Hellinger divergences (HD) at DMP positions within gene regions (**a**) or TE regions (**b**). The hierarchical clustering was built using Ward agglomeration method, and Hellinger divergence (HD) was computed by using the centroid of corresponding wild type samples. HD formula is reported elsewhere [21]

Hierarchical clustering of methylome data for individual plants from all four states in Arabidopsis used methylation level changes (computed as Hellinger divergence) at intragenic DMPs. The result produced clustering of individual plants (biological replicates) from the same population, with plants from different states separating to 6 branches distinct from WT control groups (Fig. 4). Despite originating from the same cross, Epi 8 and Epi 24 separated to two subgroups (Fig. 4a), in agreement with their distinct phenotypes (Fig. 2). The data suggested that Epi 24 genic DNA methylation repatterning was more closely related to that of graft progeny (HEG; state 3) than to its full-sib Epi 8 (Fig. 4a). Epi 10 and Epi 19 also formed two closely related clusters consistent with their phenotypic similarity. In contrast, hierarchical clustering of transposable element-associated DMPs in the four epi-lines produced outcomes in keeping with lineage, so that Epi 8 and Epi 24 now clustered together, as did Epi 10 and Epi 19 within a single sub-cluster, and both clusters separated from the graft progeny (HEG) state 3 (Fig. 4b). Thus, gene-associated methylation repatterning could be discerned to reflect plant phenotype among lines that are genetically uniform.

### Methylome data support epi-line revertants as closely related to *msh1* memory state 2

We included Epi-line (state 4) revertant samples in Arabidopsis in methylome analyses to test for evidence of methylation repatterning in revertants versus epi-line full-sib samples. Principal component with linear discriminant analysis (PCA-LDA) of these datasets using Hellinger divergence produced distinct clustering of epi-line (non-revertant) from revertant individuals deriving from the same progeny population. The epigenomic features that distinguished revertant from epi-line full-sib progeny presumably reflect inherent epigenomic stochasticity. Revertant methylome datasets showed a closer relationship with data from *msh1* memory state 2 (Fig. 3b). Specifically, of 2192 differentially methylated genes (DMGs) identified in the memory state 2 dataset, 1536 (70%) were shared with the revertant dataset, where overlap with a non-revertant epi-line was 1227 (55%) DMGs.

The *msh1* memory plants were derived from an independent population, so the similarity detected is expected to be meaningful rather than merely the consequence of lineage. A relationship of the revertant methylome profile with *msh1* memory would be consistent with their demonstrated similarity in plant phenotype (Fig. 3). These results support previous indications of *msh1* memory as distinctive in methylome features [18] and confirm low-frequency conversion of epigenetic state 4, enhanced in growth vigor, to something resembling the state 2 persistent stress response phenotype.

### Epi-line methylome datasets are altered in growth-related gene networks

**Fig. 5:**
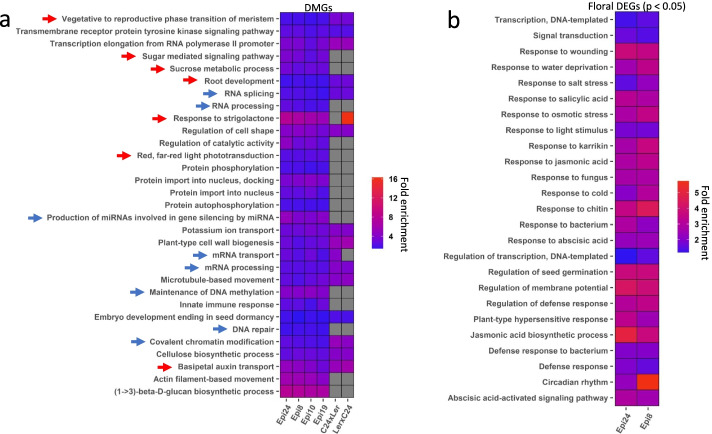
Significant enriched GO pathways by DMG and DEG analysis in epi-lines. **a** Enriched GO pathways shared by the four epi-lines in Arabidopsis as well as by F1 hybrid DMGs from the C24 x L*er* cross by our analysis. Original methylation data for the F1 hybrid were previously reported [22]. Heat map was generated using the fold enrichment of GO terms (FDR < 0.05) common between all four epi-lines. Blue arrows indicate pathways likely contributing to epigenetic change and red arrows indicate pathways likely associated with plant developmental changes. Complete list of GO terms is available in  Extended Data 2 and 3. **b** Enriched GO pathways from DEGs (FDR<0.05) shared by Epi 8 and Epi 24 floral stem tissues. Heat map displays the fold enrichment of GO terms (FDR < 0.05) common between both the epi-lines. Complete list of GO terms is available in Extended Data 4. DAVID GO (version 6.8) [23] was used for GO enrichment analysis. GO terms with EASE score (a modified Fisher exact *P*-value) < 0.05 were used for FDR calculation. FDR was calculated using package stats (version 3.6.0; p.adjust method = FDR) in R. Package ggplot2 (version 3.3.3) in R was used to generate heatmap

To investigate the relationship of intragenic differential methylation to emergent phenotypes, we focused on the identification of differentially methylated genes (DMGs). DMGs for each epi-line population were identified by applying generalized linear regression analysis (GLM) to test significance of the difference between group DMP counts (WT vs. epi-lines) in genes. This analysis identified 3204, 2860, 3208, and 2797 DMGs in Epi 8, Epi 10, Epi 19, and Epi 24, respectively (Additional file 2), with significant overlap between the epi-line datasets (Fig. S4). To investigate coincidence of DMG functional relationships in each population, we conducted a gene network-based enrichment analysis. DMGs from the different epi-lines shared enrichment for specific functional networks, particularly epigenetic-related processes (blue arrows) and plant developmental pathways (red arrows) (Fig. 5a; Additional file 2). For example, we detected enrichment for pathways involved in covalent chromatin modifications, DNA repair, maintenance of DNA methylation, RNA splicing and processing, and production of miRNAs involved in gene silencing. Toward plant development, we identified pathways for vegetative to reproductive phase transition, sugar metabolism and signaling, response to strigolactone, root development, auxin transport, and red/far-red light phototransduction. These outcomes reflect a non-random quality of DMP datasets, implying that methylation machinery-targeted gene loci and their respective networks can be identified based on methylome signal.

We analyzed methylome data previously reported for the Arabidopsis F1 heterotic cross of ecotypes C24 and L*er *[22] with the methylome analysis methods that were applied to the *msh1* datasets (Additional file 3). The enriched networks emerging from F1 hybrid (C24 x L*er*, and L*er* x C24) data showed partial conformity to what was identified in *msh1*-derived epi-line data, emphasizing many of the same developmental pathways (Fig 5a). DMG overlap between hybrid data and epi-lines ranged from 47% overlap with Epi 10 to 54% overlap with Epi 8. This apparent alignment of methylome-identified gene pathways between the heterotic hybrids and *msh1* epi-lines supports linkage of phenotype with methylome repatterning.

Examination of gene transcript changes in Arabidopsis epi-line populations involved sampling floral stem tissues, where MSH1-expression is enriched [13, 24]. Epi 8 and Epi 24, selected as full-sib lines differing in plant phenotype, were compared by RNAseq analysis of floral stem tissues. Our primary purpose in deriving gene transcript abundance data was to derive differentially expressed gene (DEG) datasets that could be integrated with methylome data for more robust gene network analysis. We have shown in previous studies that the direct overlap between DEG and DMG datasets is limited, with DMGs emphasized in upstream regulatory components and DEG data often identified in downstream components of a pathway [18, 19]. Epi 8 and Epi 24 analysis revealed 1375 DEGs from floral stem in Epi 8 and 1033 for Epi24 (*p* < 0.05; FDR < 0.05; Additional file 4).

To assess the impact of tissue type in gene expression changes, we also compared Epi 8 and Epi 24 root DEG datasets and included Epi 8 leaf DEG data to compare Epi 8 leaf, root, and floral datasets (Additional file 1: Fig. S5, Additional file 4). GO pathways identified in Epi 8 and Epi 24 root samples were consistent with those identified in floral stem samples and showed relatively few differences between the two lines except for notable enrichment in defense response-related pathways evident in Epi 8. Comparison of Epi 8 root and leaf samples showed enrichment for root morphogenesis and development pathways in the root DEG dataset, and differential representation of circadian and light response pathways. These tissue comparisons suggested that a feature distinguishing the Epi 8 and Epi 24 DEG datasets was Epi 24 enrichment in defense pathways in root tissues, whereas Epi 8 showed this enrichment in leaf tissues. Greatest DEG overlap occurred between root samples or between leaf and floral stem samples (Additional file 1: Fig. S5b), with DMG datasets overlapping to the greatest extent with floral stem DEG data (Additional file 1: Fig. S5c).

Network enrichment analysis of derived floral stem DEG datasets revealed that potential pathways that are altered in response to *msh1* effects in floral stem emphasize environmental responsiveness (Fig. 5b). Several of the pathways identified in DEG data appear to reside downstream to those identified by methylome data for gene regulation and development. These DEG-associated pathways involved abiotic and biotic stress responses, circadian rhythm- and phytohormone response-related networks. Regulation of transcription was also prominent. Integration of DEG and DMG datasets served to amplify the *msh1*-associated network enrichments relative to either dataset alone.

### Differential methylation and expression analysis identified putative central gene hubs for the epi-line state

To investigate the interaction of DMG and DEG datasets in epi-lines involved inputting DMG and DEG data to Cytoscape (3.8.2) to construct protein-protein interaction (PPI) maps, followed by *k*-means cluster analysis to identify putative core networks carrying central gene hubs. A *k*-means cluster machine learning algorithm uses betweenness centrality, closeness centrality, average shortest path length, clustering coefficient, degree, and eccentricity as parameters, allowing the identification of clusters that contain the most centralized nodes (proteins) in the PPI network.

**Fig. 6:**
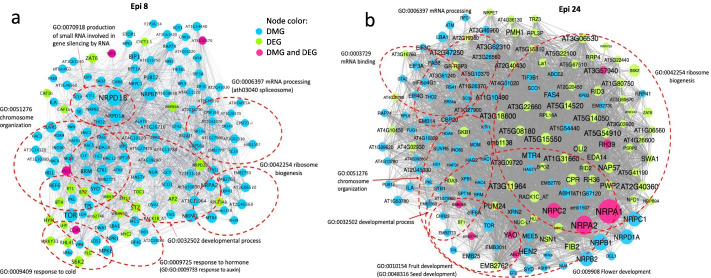
PPI hubs derived from subsets of network-related DMGs and DEGs in Epi 8 and Epi 24. Epi 8 and Epi 24 represent progeny lines derived from the same cross, with Epi 8 displaying enhanced vegetative growth rate and Epi 24 significantly enhanced seed yield. The main subnetwork of hubs was obtained with the application of machine learning *k*-means clustering on the set of 3647 (Epi 8, panel **a**) and 3523 (Epi 24, panel **b**) network-related DMGs and DEGs (*p* < 0.1) identified in the Arabidopsis epi-line vs WT comparison. The analyses yielded 153 (**a**) and 346 (**b**) hub genes to form a closely related subnetwork. GO network enrichment analysis from the string application in Cytoscape [27] was used to identify enriched gene function pathways (FDR < 0.05) within the network. Blue represents DMGs, green represents DEGs, and magenta represents both DMGs and DEGs. Size of each node is proportional to its value of node degree and label font size is proportional to its betweenness centrality

In Arabidopsis Epi 8, a total of 3647 unique loci from DMGs and DEGs were used in the analysis to yield a PPI network formed by 430 genes. Functional enrichment analysis (FDR < 0.05) of these putative hub genes with the STRING [25] database functional enrichment tool revealed a PPI network of 153 hub genes and associated functional networks (Fig. 6a). These core networks included developmental process, response to hormone (particularly auxin), response to cold, chromosome organization, mRNA processing, spliceosome, and ribosome biogenesis. Comparison to Epi 24, with a total of 3523 unique loci from DMGs and DEGs forming a much larger PPI network of 346 genes, showed that several of these same processes were represented (Fig. 6b). However, Epi 24 analysis revealed more prominent changes in development-related gene expression and, most notably, a robust and overlapping effect in ribosome biogenesis-related gene expression (Fig. 6b). Ribosome biogenesis is integral to growth response [26]. Because Epi 8 and Epi 24 are full-sib populations from the same cross, these differing features are predicted to relate to their distinct phenotypes, with Epi 8 enhanced in vegetative growth and Epi 24 showing significantly increased reproductive vigor (Fig. 2).

### Epi-state comparisons in Arabidopsis reveal conserved *msh1* epigenome targets within biologically meaningful gene networks

**Fig. 7:**
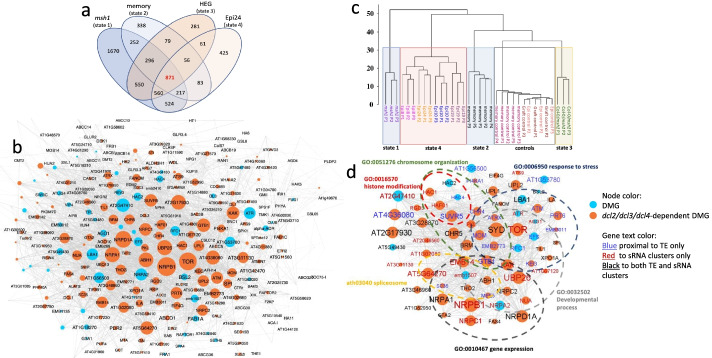
The relationship of 871 core hub genes to *msh1*-derived states and biologically meaningful core networks. **a** Venn diagram of Arabidopsis DMGs identified from four different *msh1*-derived states (Col-0 genetic background): *msh1* mutant (state 1), *msh1* memory (state 2), graft progeny (HEG, state 3), and epi-line (Epi 24, state 4). **b** An overview of the PPI networks and individual 871 core hub genes. Blue represents DMGs, orange represents the DMGs that are *dcl2/dcl3/dcl4*-sensitive in graft progeny contrasting mutant rootstock analyses. **c** Hierarchical clustering of individual plant datasets from four different *msh1-*derived states based on the sum of Bayesian methylation level difference of DMPs over the 871 core genes from panel **a**. The hierarchical clustering was built using Ward agglomeration method. The Bayesian methylation level difference was computed as described previously [18]. **d** Main subnetwork of hubs obtained with the application of a machine learning *k*-means clustering algorithm on the set of 871 core genes from panel **a**. GO network enrichment analysis from the string application in Cytoscape [27] was used to identify enriched gene function pathways (FDR < 0.05) within the network. In total, 67 genes involved in enriched networks were identified. Blue nodes represent DMGs, with orange representing the DMGs that are *dcl2/dcl3/dcl4*-dependent in graft progeny contrasting mutant rootstock analysis. This 67-gene set supplied the RdDM candidate target genes for further study. Blue gene text represents DMGs proximal to only TE sequences, red text designates genes that are proximal to only sRNA clusters, and black text represents genes proximal to both TE and sRNA clusters. For both **b** and **d**, the size of each node is proportional to its value of node degree and the label font size is proportional to its betweenness centrality

We attempted to better define the relationship of genic methylation repatterning among the four distinct *msh1*-derived states by first assessing DMG overlap. Figure 7a shows that 871 DMGs were shared among the four *msh1* states (Additional file 5), comprising 17.6% of *msh1* (state 1), 39.7% of memory (state 2), 31.6% of graft progeny (HEG, state 3), and 31.1% of epi-line (state 4, Epi 24) DMGs. The overlap established a conserved *msh1* “core” DMG dataset for further analysis (Fig. 7a). Each state was also classified by state-specific DMGs, accounting for 33.8% in state 1, 15.4% in state 2, 10.2% in state 3, and 15.2 % in state 4 (Fig. 7a). Of the 871 “core” *msh1*-associated DMGs, 531 (61%) could be placed within known networks (Fig. 7b).

We previously showed that components of the RdDM pathway were necessary for induction of *msh1* state 1, transition from state 1 to state 2, and generation of state 3 following grafting [18, 19]. Therefore, we incorporated RdDM mutants to functionally identify RdDM-targeted DMGs within this core dataset. We contrasted methylome datasets for *msh1* (state 1) versus *dcl2/dcl3/dcl4/msh1* quadruple mutant and graft progeny from Col-0/Col-0_*msh1*_ (state 3) versus Col-0/Col-0_*dcl1/dcl3/dcl4/msh1*_ grafts to catalog DMGs that were RdDM (sRNA)-dependent [19]. These subtractive datasets confirmed that 674 (77%) of the 871 core DMG loci were predicted to be *DCL2,DCL3,DCL4*-dependent by obtaining the overlap between the 871 DMG core dataset and the *msh1* vs *dcl2/dcl3/dcl4/msh1* and graft datasets (Additional file 5). Figure 7b shows the PPI core hub network of DMGs with *DCL2,DCL3,DCL4*-dependent colored orange. These outcomes are evidence of a relationship between *msh1*-directed epigenetic effects and RdDM pathway influence on targeted methylome repatterning.

Whereas 871 core DMGs were shared among the four *msh1* states, the methylation changes within these 871 loci also served to discriminate the four states. Figure 7c shows the results of hierarchical clustering for individual plants from the four epigenetic states with separation to four distinct clades by using only DMP methylation information over the 871 *msh1* core genes. This clustering relationship helped validate our selection of a single memory (state 2), graft progeny (state 3), and epi-line (Epi 24, state 4) dataset for the identification of the overlap. The four epi-lines produced highly related datasets (Additional file 1: Fig. S4). Based on the PPI network derived for the 871 DMGs, we conducted *k*-means clustering to identify putative central hubs and conduct functional enrichment analysis. Figure 7d shows the resulting network of 67 candidate hubs (Additional file 7) involved in response to stress, developmental (growth) process, gene expression, spliceosome function, histone modification, and chromosome organization networks. Over 80% of the 67 hubs were predicted to be RdDM targets, shown in orange, with several central players in plant development and environmental stress response, including the cell growth regulator *TARGET OF RAPAMYCIN* (*TOR*), the SWI2/SNF2-like, meristem identity gene *SPLAYED* (*SYD*), its close homolog *BRAHMA* (*BRM*), and the chromatin remodelers *PICKLE* (*PKL*) and *PICKLE-RELATED 1* (*PKR1/CHR4*).

Network-based analysis outcomes can be influenced by the available gene annotation and gene relationship information for any given species. To independently evaluate gene associations identified in these network-based studies, we performed weighted correlation network analysis [28, 29] with the 871 core *msh1*-associated DMGs. This alternative approach used pairwise comparisons within a multi-dimensional dataset to define clusters that could be assessed for network features in the absence of gene annotation data. At a correlation coefficient of 0.5, we identified 300 connected genes, with 44 (66%) overlapping with the 67 hub loci identified by *k*-means clustering (*p*-value = 0.01015 Fisher exact test). At a correlation coefficient of 0.6, we identified 166 genes, with 33 (49%) overlapping with the 67 hub loci (*p*-value = 0.000333 Fisher exact test) (Additional file 1: Fig. S5). These data reflect meaningful associations between the identified 67 hub loci for their methylation content and the discriminatory power of such information to distinguish phenotype among the four states.

### Transposable elements and sRNAs associate with candidate RdDM target loci among the 871 *msh1* DMGs

The RdDM pathway actively targets transposable element (TE) sequences [6, 7], prompting investigation of TE association with *msh1*-responsive loci. Looking at the 871 DMGs common among *msh1* states, we detected association of these loci with TEs and sRNA (20-24 nt) clusters that was higher than genome-wide levels (61% DMGs within 2 kb of TE vs. 47% genome-wide; 77% DMGs within 2 kb of an sRNA cluster vs. 46% genome-wide). This enrichment for TE and sRNA cluster proximity increased further when we subset the *dcl2,dcl3,dcl4*-sensitive DMGs (65% DMGs within 2 kb of TE; 81% DMGs within 2 kb of an sRNA cluster). Yet, when we focused on the 67 hub DMGs derived by *k*-means clustering, only sRNA cluster enrichment was seen (49% of DMGs within 2 kb of TE; 72% DMGs within 2 kb of an sRNA cluster) (Additional file 6). Figure 7d shows only TE-associated genes (7) in blue text, only sRNA-associated genes (22) in red text, and genes associated with both TE and sRNA (26) in black text. Hence, although TEs may influence the methylation status of proximal genes and act as RdDM targets, sRNA cluster association regardless of TE proximity could define RdDM targets for the *msh1* effect.

We found possible evidence of association between TE family and DMG proximity (Additional file 6). Comparing the number of members in TE families between observed and expected revealed significant overrepresentation in L1 and Gypsy families and underrepresentation in the Helitron family, both in the 871 DMGs common between *msh1* states and 674 DMGs sensitive to *dcl2,dcl3,dcl4* (Additional file 6). Further investigation is needed to reveal any biological significance of these associations. Based on the various analyses, four criteria served to classify RdDM target loci in our study (Fig. 7d): Positive loci were (1) confirmed DMGs in all four *msh1* states that (2) comprised putative network hubs based on *k*-means cluster analysis, with genetic evidence for (3) *dcl2,dcl3,dcl4*-sensitivity, and/or (4) association with sRNA clusters (Additional file 6).

### Detailed methylation analysis of selected RdDM target genes in the four different nongenetic states reveals sequence motifs encompassing *dcl2/3/4*-sensitive DMPs

Annotations of the 67 candidate hub loci supported their relevance to phenotype effects observed in the four *msh1* states. In addition to gene networks for altered gene expression and chromatin behavior, major overlapping networks appeared to reflect the transition between stress response (Fig. 7d) that underpins *msh1* and memory stress phenotypes versus growth vigor in HEG and epi-line states. Methylation features of the 67 candidate hub loci that discriminate the four *msh1* states support a model of precisely targeted repatterning (Fig. 7c). Therefore, we investigated these loci for evidence of site-specific methylation changes.

**Fig. 8:**
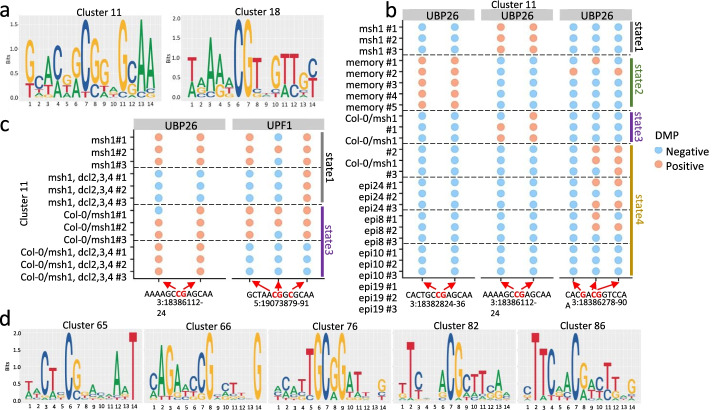
Investigation of methylation repatterning within candidate RdDM target genes that discriminate *msh1*-derived states. **a** Two of the putative cluster motifs identified based on differential gene methylation across four *msh1*-derived states. Hierarchical clustering on a set of 14-bp regions encompassing DMPs from seven (*TOR, SYD, NRPB1, NRPD1, UBP26, SUVR5, UPF1*) of the 67 core hub loci followed by DNA multiple sequence alignment of each cluster permitted the identification of methylation motifs (see “Methods” for more detail). **b** Difference of methylation levels on gene body DMPs within motif cluster 11 in the putative RdDM target gene *UBP26*. Variations on motif methylation repatterning at DMPs are shown with chromosome and position. Individual detected methylation changes are shown as color-coded dots for each plant assayed in each *msh1* state, with positive (orange) indicating DMP and negative (blue) for no detected methylation change. Each line represents a single plant dataset. **c** Sample DMPs within motif cluster 11 in *UBP26* and *UPF1* that show *dcl2/dcl3/dcl4*-sensitivity in graft rootstock (state 1) and graft progeny (state 3) from contrasted mutant rootstock experiments. Note that *dcl2/dcl3/dcl4* effects can only be assayed for *msh1* and graft progeny data. All data associated with the seven genes in this figure are shown in Extended data 7. **d** Sample putative cluster motifs identified based on differential gene methylation across four *msh1*-derived states in analyses of the 67 core hub loci followed by DNA multiple sequence alignment of each cluster for methylation motifs. A complete data report for motifs identified based on all 67 genes is shown in Additional file 8

Figure 8 and Table 2 show results of analysis for seven selected candidate RdDM target loci: *TARGET OF RAPAMYCIN (TOR)*, a regulator of cell growth, *SPLAYED (SYD)*, a chromatin remodeling component, *UBIQUITIN-SPECIFIC PROTEASE 26 (UBP26)*, required for heterochromatin silencing, *NUCLEAR RNA POLYMERASE D1A (NRPD1)*, the largest subunit of RNA polymerase IV, *RNA POLYMERASE II LARGE SUBUNIT (NRPB1)* involved in transcription*, SU(VAR)3-9-RELATED PROTEIN 5 (SUVR5)*, a gene involved in H3K9me2 deposition and gene silencing, and *UP-FRAMESHIFT1 (UPF1)*, a gene involved in RNA interference and nonsense-mediated RNA decay. These loci were selected to represent the various gene networks identified.

**Table 2:**
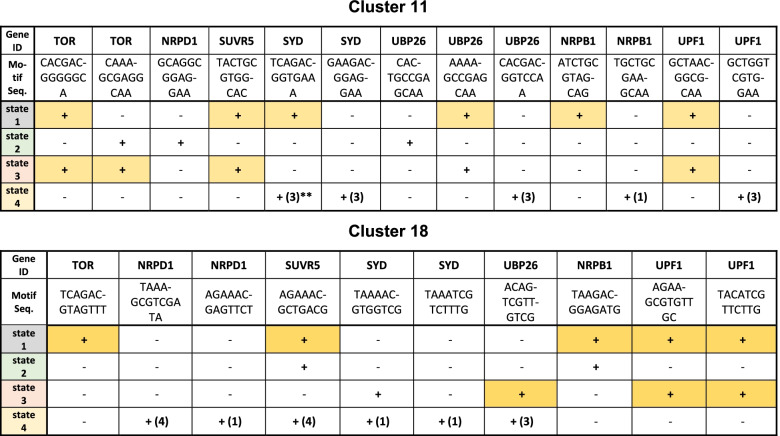
Summary data for gene methylation within motif clusters 11 and 18*

* + indicates presence of DMPs in at least two samples, − indicates absence of DMPs, color indicates *dcl2, dcl3, dcl4* sensitivity. Information about hyper/hypomethylation is available in Extended data 7

** number in parentheses refers to number of epi-lines displaying DMPs in at least 2 samples

Differential methylation analysis within the seven loci revealed evidence of state-specific repatterning (Table 2; Additional file 7). Changes in methylation at each locus were associated with identifiable sequence motifs that spanned approximately 14 nucleotides. Two sample composite motifs are shown in Fig. 8a, with examples of *UBP26* and *UPF1* state-specific methylation repatterning within cluster 11 shown in Fig. 8, panels b and c. Systematic analysis of all 67 loci revealed similar state-specific changes (Additional file 8). DMPs were assayed individually, although multiple DMPs could be detected within a single identified motif domain. DMPs were discriminated among replicated datasets, with several sites shown to be *dcl2/dcl3/dcl4*-sensitive in state 1 and state 3 (Fig. 8c; Table 2; Additional file 7). It was feasible to test *dcl2/dcl3/dcl4* sensitivity in just the two states that encompass the *msh1* rootstock [19], although these data confirm RdDM influence at single cytosine resolution. Table 2 summarizes scoring outcomes for cluster 11 and 18 motifs within the seven selected genes.

Identified DMPs did not show an obvious pattern of exon, intron or junction localization, and each gene contained multiple motif sites (Additional file 8). Evaluation of cluster motifs that encompass DMPs within the 67 *msh1* core hub loci revealed, in many cases, evidence of high-order dependencies (Fig. 8d; Additional file 8). Multiple sequence alignment (MSA) of a given DNA motif can reveal a dependence relationship between two nucleotides located at different positions within the motif, reflected in their frequencies of simultaneous occurrence. First-order dependence refers to adjacent nucleotides, typically found in CG methylation context, second-order to nucleotides spaced two nucleotides apart, and high-order to nucleotides with intervening distance of more than two nucleotides. The relationships derive from the study of Markov dependence in DNA sequences, the basis for application of hidden Markov modeling of motif findings [30].

For the motifs identified, individual consensus nucleotides were evident at variable distance from the target cytosine, positioned at nucleotide 7 on plus or minus strand within each motif. For example, the motif from cluster 65 showed invariant T at position 14 and a consensus A at position 12, while the motif from cluster 66 showed invariant G at position 14 and an AG pair consensus at positions 2 and 3, respectively (Fig. 8d; Additional file 8). In motif 76, only G resides at positions 6 and 9 with consensus T at position 5, while motifs 82 and 86 showed consensus or invariant T at position 2. These observations support the hypothesis that target methylation sites within the genes are characterized by invariant or nearly invariant sequence features.

## Discussion

*MSH1* disruption or knockdown in plants leads to aberrant recombination in both mitochondrial [14] and plastid genomes [15], with the latter triggering nuclear epigenetic changes [13, 17] that are the focus of this study. Information from these investigations, integrated with earlier data, show that *msh1*, its memory lineage, graft-derived progeny and crossing-derived epi-lines comprise distinct de novo nongenetic states. These heritable states differ phenotypically from WT, consistently displaying nascent stress response (*msh1*/memory) or growth vigor (HEG/epi-line) phenotype changes across multiple plant species. Integrating carefully designed datasets confirmed RdDM influence on dynamic and potentially adaptive phenotype plasticity in plants.

In an earlier study, we speculated that *msh1* epi-line variation in Arabidopsis was likely to be indistinguishable from graft progeny (HEG) outcomes [17]. This misinterpretation appears to be a consequence of insufficient sampling from only a single *msh1* epi-line population. In fact, DNA methylation and gene expression analysis in this study provided unambiguous discrimination of the four *msh1*-derived states on a uniform Col-0 genetic background. Yet, it was possible to identify a subset of 871 DMGs shared among the four distinct states. We interpret this finding to indicate that reprogramming events in the original *msh1* mutant are foundational to the other derived states.

The condition of *msh1* memory, and its apparent relationship to epi-line reversion, raises important questions about stable transgenerational retention of a memory state. To date, we have not observed diminishment of *msh1* memory as a whole-plant or methylome phenotype over seven sequential generations of self-pollination in Arabidopsis [18]. However, each memory generation shows evidence of DNA methylation repatterning at conserved sites [18]. This dynamic feature, together with dependence of memory induction on RdDM methyltransferase DRM2 and sRNA variation [18], suggest that transgenerational stability involves re-establishment of de novo methylation sites by inherited sRNAs each generation. Recurrent inheritance of epigenetic memory states in *C. elegans* points to small RNAs as the most likely epigenetic vector for stable transmission [31, 32] and to direct chromatin modification [33]. In *C. elegans*, two features of sRNAs facilitate their inheritance: the ability to circumvent sRNA degradation processes and to amplify by templating their own synthesis [34–36]. The extent to which sRNA transgenerational stability is sustained similarly in plants is unclear, but parental sRNA activity occurs in gametes and developing embryo [37, 38]. Epi-line (state 4) reversion back to a condition resembling *msh1* memory (state 2) implies that interaction of the *msh1* memory genome with naïve (WT) through reciprocal crossing does not impede reformation, at low frequency, of intact memory in subsequent generations. These sustained memory epigenomic features are presumed to be sRNA-directed.

Incorporation of analysis methods that discriminate treatment-associated intragenic DMP variation [21] provided sufficient resolution to conduct gene network-based analysis. A similar application of these methods to contrasting datasets from *msh1*, *dcl2/dcl3/dcl4/msh1*, and the corresponding graft-derived progeny functionally identified candidate RdDM target genes. Target loci were derived based on their classification as network hubs, differential methylation in all four *msh1* states, DCL2/DCL3/DCL4-dependent effects, and/or association with sRNA clusters. Data from this analysis, which could not be accomplished using traditional DMR-based methods [18, 21, 28], opens the door to investigation of plant environmental or heterotic response as a precise and predictable epigenomic phenomenon.

Candidate RdDM targets identified in the study represent prominent members within pathways relevant to epigenetic regulation and plant adjustment to environmental change. The known function of these loci aligned with observed *msh1-*derived phenotypes. *msh1* and its memory lineage display complex stress phenotypes, altered expression of abiotic and biotic stress pathways and circadian clock components, and suppressed growth rate and delayed flowering [16, 18, 39]. Graft [19] and epi-line progeny effects include changes in phytohormone and environmental response pathways and heterosis-like gene expression.

As anticipated, we found only modest overlap between the 67 core hub loci associated with *msh1* effects and DEG data; this outcome is common to such studies. RNAseq analysis was conducted with samples from pooled cell types, which confounds detection of spatio-temporal expression, and does not reveal changes in transcription rate or splicing activity. Of the 67 core hub loci, at least 43% (29) show cell-specific differential expression in the absence of *MSH 1 *[24], and 66% (44) exhibit cell-type specific expression in the inflorescence [40]. Instead, our analysis focused on integration of gene pathways identified in common by DMG and DEG data, permitting greater resolution of these pathways than was achieved by either dataset alone. We have shown previously with this type of integrated pathway analysis that DMGs tend to represent upstream regulators with DEGs more abundant as downstream pathway components [18, 19]. In this study, our analysis permitted detection of the more robust ribosome biogenesis pathway component within datasets from epi-lines with greater growth vigor. A majority of this enhanced pathway was comprised of DEGs, several essential to plant growth and development (e.g., *ROOT INITIATION DEFECTIVE 3* (*RID3*)), organ size control and cell proliferation (e.g. *OLIGOCELLULA2* (*OLI2*)), mitotic division cycles and gametogenesis (e.g., *SLOW WALKER 1* (*SWA1*)), and active cell division (e.g., *BLOCK OF CELL PROLIFERATION 1* (*BOP1*)). We suspect that these DEGS operate downstream to central growth regulators.

Methylome-derived gene network information revealed a surprising number of genes likely to be integral to the observed *msh1*-triggered “pivot” from stress to growth vigor phenotypes. For example, *TARGET OF RAPAMYCIN* (*TOR)*, *SPLAYED* (*SYD)*, and *BRAHMA* (*BRM*) emerged as putative RdDM target loci that undergo differential methylation in *msh1*-derived states. TOR kinase serves as a key developmental regulator in the plant that directs growth by integrating nutrient and environmental signals [41–43]. TOR functions antagonistically to stress response pathways and is postulated to serve as a decision point in the plant’s energy allocation for defense versus growth [43]. TOR influences plant growth and yield and acts to regulate the circadian clock in plants [44, 45]. In animal systems, TOR is known to also function within the nucleus, where it triggers chromatin and epigenomic responses [46]. These features suggest that TOR may be intrinsic to *msh1* developmental reprogramming.

SPLAYED is a SWI2/SNF2-like protein in the SNF2 subclass that acts to regulate shoot apical meristem identity. SYD functions in meristem maintenance and regulates several early developmental processes [47–49]. BRAHMA is also a SWI2/SNF2 ATP-dependent chromatin remodeler and a SYD homolog; both mutants display pleiotropic phenotypes. *BRM* and *SYD* appear, based on mutant phenotypes and genome-wide occupancy studies, to carry out both redundant and distinct functions [48]. Further, *SYD* and *BRM* can act antagonistically with Polycomb-group (PcG) proteins [49]. For example, *BRM* promotes vegetative growth by suppressing PcG-associated H3K27me to upregulate the *SHORT VEGETATIVE PHASE* flowering repressor [50]. Likewise, *BRM* is required to establish epigenetic heat stress memory in association with *FORGETTER 1 *[51]. In genome-wide occupancy studies, SYD directly targets nearly 6000 mainly developmental and stress-responsive genes in Arabidopsis, influencing H3K27me3 levels at several target loci [49]. Consequently, these genes serve as innate regulators of plant vegetative and reproductive growth.

In the context of the *msh1* system, modulation of these *msh1* RdDM targets could influence the plant growth program for heightened stress response versus growth vigor, as well as epi-line vegetative growth vigor versus reproductive growth variation. A fourth chromatin remodeling component, PICKLE (PKL), similarly identified as an RdDM target in *msh1*, functions to maintain RdDM-targeted methylation [52]. PKL acts synergistically with RdDM pathways to influence plant development [53].

Transition from *msh1* “stress” to “growth” state entails interaction of the *msh1*-modified nuclear genome with naïve (WT) achieved through crossing or grafting. Similar epigenomic interaction between epigenetically modified and native states is implicated in heterosis [54, 55]. Manipulation of the *msh1* system leads to a condition that resembles classic F1 heterosis, but differs in its subsequent heritability.

What is striking about a majority of *msh1* RdDM target genes is their broad regulatory capacity in stress response and development, interpreted to reflect the vital role of integrated signaling and metabolic networks to calibrate environmental response in plants [56]. In this study, TE methylation data aligned according to lineage relationships, whereas gene methylation agreed with phenotype changes in epi-line comparisons. This observation further links RdDM effects on intragenic methylation with possible plant phenotype modulation. Identification of loci based on site-specific methylation suggests that adjustments in chromatin poise, or amenability to expression, can involve precise methylation changes. These gene methylation effects can facilitate alternative transcript splicing behavior in response to cellular cues [57] that would not likely be detectable in DEG datasets. The 871 DMGs shared across *msh1* states, and their 67 core hub loci, include significant RNA spliceosome pathway components (Fig. 7d).

The ability to predict sites of differential gene methylation within major regulatory and chromatin remodeling genes serves to mark these loci during environmental response or growth changes in the plant. Results suggest the existence of methylation motifs with variable order interdependency of specific motif nucleotide positions. Overall, motif consensus sequences showed statistically significant departure from randomness, supporting the participation of DNA features in local epigenomic behavior [58]. We expect that the high-order dependencies observed within methylation motifs reported here can be captured by future deep neural network machine learning models [59, 60]. Moreover, the MSH1 system, as a biological model, can generate sufficiently robust methylation motif libraries to feed such machine learning predictive models. We consider the system of intragenic DMP motifs described in this study to reflect a previously uncharacterized fine-tuning mechanism of epigenomic regulation, in contrast to the high-density DMR changes that characterize RdDM-mediated TE and promoter silencing [61]. Epigenomic effects reported here should prove valuable to deciphering environmentally influenced complex trait expression.

## Conclusions

The dynamic nature of DNA methylation during developmental and environmental changes renders elusive the essential features of methylome variation. Analyses based on changes in methylation density and magnitude are less informative for phenotype-associated methylome interpretation than are studies of low-density, intragenic methylation repatterning. The MSH1 system provides unique materials for these initial efforts toward methylome decoding, identifying, and confirming RdDM gene targets within networks that undergo epigenomic reprogramming during growth-versus-stress response transitions. The extent that information from this study extrapolates across plant species will have important implications for understanding genotype x environment influences on plant fitness.

## Methods

### Plant materials and growth conditions

For *Arabidopsis thaliana* material used in the study, seeds (Col-0 genetic background) were sown on peat mix in square pots, with stratification at 4 °C for 2 days before transfer to growth chambers (22 °C, 12 h DL, 120−150 μmol m^−2^ s^−1^ light). To generate epi-lines, two third-generation *msh1* T-DNA mutant (SAIL_877_F01) plants were used to pollinate Col-0 to generate two independent F1 populations. Derived F1 progenies were self-pollinated to generate F2 populations that were genotyped for the *msh1* T-DNA mutation. *MSH1* F2 plants were self-pollinated to produce F3 families that were used in the study.

### Phenotypic data

For leaf area, photos of trays containing randomly placed treatment and control plants were taken at 33–34 DAP using a Canon EOS DIGITAL REBEL XSi camera. Total leaf area was measured using ImageJ (v1.51j8; https://imagej.nih.gov/ij/index.html) software. For root-related analysis, seeds were sown in square pots containing sand media topped with standard peat mix to allow seed germination. At 33 DAP, roots were cleaned under running water to remove sand particles and allowed to dry at room temperature for at least 3 weeks before measuring dry weight. At the same time, aboveground rosettes were also preserved, cleaned, and dried for dry leaf weight measurements.

### Whole-genome bisulfite sequencing and DNA methylation analysis

In Arabidopsis, Col-0 WT, Epi 8, Epi 10, Epi 19, and Epi 24 plants were grown together as one experiment, and WT, epi-line revertant, and epi-line non-revertant plants were grown together as a second experiment, with three plants from each line selected for sequencing. For the reversion experiment, an F3 population obtained through Col-0 WT x *msh1* memory was selected based on evidence of reversion activity. From this population, revertant and non-revertant full-sibs and a 4th generation memory sample in triplicate were used for bisulfite sequencing. Whole rosettes at the bolting stage from three biological replicates were flash frozen and ground in liquid nitrogen.

Tissues were ground and processed with the DNeasy Plant Kit (Qiagen, Germany) for genomic DNA with RNA removal according to the manufacturer’s protocol for subsequent bisulfite sequencing. Whole-genome bisulfite sequencing was conducted on the Hiseq 4000 (Illumina, USA) at BGI-Tech (Shenzhen, China) and Novogene (Beijing, China), according to the manufacturer’s instructions. Then, 150 bp read length and at least 4 Gb data per sample were derived for Arabidopsis samples and at least 20 Gb per sample was collected for tomato samples. Raw sequencing reads were quality-controlled with FastQC (version 0.11.5), trimmed with TrimGalore (version 0.4.1) and Cutadapt (version 1.15), then aligned to the reference genome using Bismark (version 0.19.0) with bowtie2 (version 2.3.3.1). DMPs were identified using Methyl-IT (version 0.3.2) R package as described previously [18, 19, 21]. Briefly, Hellinger Divergence (HD) was calculated with a pool of control (wild type) samples as reference as described previously [21]. Cytosines with methylation level difference >20% in the treatment vs. reference comparison were selected and further filtered by estimating the optimal cutoff for HD based on a machine learning approach to obtain DMPs (https://genomaths.github.io/methylit/). To identify the DMGs, we selected loci with at least seven DMPs and minimum DMP density of 3 per 10 kbp, followed by group comparison using likelihood ratio test (LRT) to select loci with log2fold change >1 and adjusted *p*-value < 0.05 (Benjamini and Hochberg method). TAIR10 version 38 GTF was used for Arabidopsis annotation. DAVID Bioinformatics Resources 6.8 was used for GO function enrichment analysis. GO terms with EASE score (a modified Fisher exact *P*-value) < 0.05 were selected and further used for FDR calculation. FDR was calculated using package stats (version 3.6.0; p.adjust method = FDR) in R. Package ggplot2 (version 3.3.3) in R was used to generate heatmap.

For hierarchical clustering, we used the *hclust* from the R package stats (version 3.6.0). Either sum of Hellinger divergence (HD) or sum of methylation level over a given locus was used for analysis. A complete documentation and package (with Methyl-IT) is available at https://genomaths.github.io/.

### RNA sequencing and analysis

For the Arabidopsis WT and epi-lines, floral stem tissues from 3 individual 6-week-old plants of epi24, epi8, and WT; root tissue from 3 individual 10-day-old plants of epi24, epi8, and WT; leaf tissues from 3 individual 4-week-old plants of epi8 and WT were flash frozen and ground in liquid nitrogen. Tissues were subjected to RNA extraction, including DNA removal, using the NucleoSpin RNA Plant Kit (Macherey-Nagel, Germany). sRNA extraction used the NucleoSpin miRNA Plant Kit (Macherey-Nagel, Germany) following the manufacturer’s protocol. RNA libraries were constructed as described in the TruSeq RNA Sample Preparation v2 Guide and were sequenced with the 150 bp read, paired-end option, in the Novaseq analyzer (Illumina, USA) at Novogene (Beijing, China). Raw sequencing reads were quality-controlled, trimmed with TrimGalore (version 0.4.1), and then aligned to the TAIR10 reference genome using STAR (version 2.7.3a) with –twopassMode = Basic and –outFilterMultimapNmax = 1 parameters, retaining only uniquely mapped reads. QoRTs software package (version v1.3.0) with –minMAPQ = 25 option were used to generate BAM files. edgeR package (version 3.26.8) was used for gene count normalization and to identify DEGs (*p* < 0.05, FDR < 0.05, |log2FC| ≥ 0.5). DAVID Bioinformatics Resources 6.8 was used for GO function enrichment analysis. GO terms with EASE score (a modified Fisher exact *P*-value) < 0.05 were selected and further used for FDR calculation. FDR was calculated using package stats (version 3.6.0; p.adjust method = FDR) in R. Package ggplot2 (version 3.3.3) in R was used to generate heatmap.

### Core hub PPI networks, functional enrichment, and weighted correlation networks

Cytoscape (ver. 3.8.2), and the built-in application STRING, clusterMaker was used to construct the core hub PPI network. The input DMGs and DEGs were placed into the STRING data network by Analyze Network function, and then classified into 3 clusters by using *k*-means cluster function, with Euclidean distance as the distance metric, betweenness centrality, closeness centrality, average shortest path length, clustering coefficient, degree and eccentricity as node attributes, and 300 iterations were carried out. Genes in the cluster highest overall centrality were selected as core hubs and subject to functional enrichment analysis using the string enrichment function (FDR < 0.05).

To confirm network associations, we conducted weighted correlation network analysis. Individuals were represented as vector of genes of 600 (67)-dimensional space of DMGs, where each gene was represented by the sum of Hellinger divergence at each DMP on the gene body. Next, principal component analysis (PCA) was applied to identify the genes with the highest contributions to the discrimination between the four *msh1* states and controls. PCA is a standard statistical procedure to reduce data dimensionality, to represent the set of DMGs by new orthogonal (uncorrelated) variables, the principal components (PCs) [62], and to identify the variables with the main contribution to the PCs carrying most of the whole sample variance. In the current case, components carrying 1% of the sample variance (Guttman-Kaiser criterion [63]) and accumulating at least 95% of the whole sample variance were selected. Next, genes were represented as vectors of principal components, where vector (gene) coordinates are PC loadings. A PC score (genes-core) was computed as the Euclidean norm of the vector of principal components. Since the sum of the squared of variable loadings over a principal component is equal to 1, the squared loadings tell us the proportion of variance of the given principal components explained by the gene. Thus, the greater the PC score value, the greater the fraction of cumulative variance from the whole sample variance is carried by the gene and, consequently, the greater the discriminatory power carried by the gene.

The sum of *HD* on the gene body was computed with Methyl-IT function *getGRegionsStat* and the principal component with function *pcaLDA*, which applies the PCA calling function *prcomp* from the R package “*stats*.”

### Motif clustering

Small regions (14 bp) encompassing DMPs in at least three samples were identified and considered as DNA methylation motif candidates in the 67 identified *msh1* core hub genes. A distance matrix was estimated on the set of selected regions using function *dist.dna* from *ape* R package (version 5.5). Hierarchical cluster analysis on the set of selected regions (using the previous estimated distance matrix) was accomplished with function *hclust* from *stats* R package (version 4.1.1) and grouped to 100 clusters. UPGMA approach was applied as agglomeration algorithm. Clusters with fewer than 10 regions where discarded. A DNA multiple sequence alignment on each cluster of sequences was accomplished with MUSCLE algorithm implemented on Bioconductor R package *muscle* (version 3.14). The sample motifs presented in Fig. 8 resulted from the specific analysis of genes TOR, SYD, NRPB1, NRPD1, UBP26, SUVR5, and UPF1.

#### Motif score

We defined the motif score *s*_*jk*_of the aligned sequences *j* and *k* of length *N* as the logarithm base 2 of the number of matched bases found in the alignment. Formally:

1 $${s}_{jk}={\mathit{\log}}_2\sum_{i=1}^N{I}_{jk}^i$$

where $${I}_{jk}=\left\{\begin{array}{c}1\ if\ {b}_j^i={b}_k^i\\ {}0\ \mathrm{otherwise}\end{array}\right.$$ for every base position *i* on sequences *j* and *k*. The maximum motif score is then: *Max*{*s*_*jk*_} = *log*_2_*N*. The motif score in a MSA is, thus, defined as:

2 $$S=\frac{1}{m}\sum_{j=1}^{M-1}\sum_{k=j+1}^M{s}_{jk}$$

For a MSA with *M* sequences of length *N* each, the number of pairwise comparisons is: $m=\frac{M\times \left(M-1\right)}{2}$. For a fixed value of motif length *N*, the perfect MSA of DNA sequence motifs would have the maximum score: *Max*{*S*} = *log*_2_*N*. In other words, the maximum amount of information carried on an MSA in this modeling is *I*_*max*_ = *log*_2_*N*, and the amount of information (the uncertainty change) carried by a MSA is given by the expression:

3 $$I=-\left(S-{\mathit{\log}}_2N\right)$$

The same result is obtained if the letter frequencies in the MSA and Shannon entropies, before (perfect alignment) and after, are estimated instead of the number of matches. Then, the alignment information is computed as: *I* =  − (*H*_after_ − *H*_before_) [64].

#### Monte Carlo testing of MSA randomness

A Monte Carlo test (MCT) of how a given DNA multiple sequence alignment differs from randomly generated MSAs was accomplished assuming:

1. The count vectors *n*_*k*_ summarizing the observed DNA base frequencies in *N* column (*k* = A, C, G, T) are distributed according to the multinomial distribution with parameters *p*_*k*_.
2. In Bayesian framework, since Dirichlet distribution is the conjugate prior distribution of the multinomial distribution, the parameter vector *P* = (*p*_*A*_, *p*_*C*_, *p*_*G*_ , *p*_*T*_) is drawn from a Dirichlet distribution ${\mathcal{D}}_{\alpha P}$ with parameter vectors 𝛼 = (𝛼_1_, 𝛼_2,_ 𝛼_3_, 𝛼_*4*_) and *Q* = (*q*_*A*_, *q*_*C*_, *q*_*G*_ , *q*_*T*_).

A *N* × 4 matrix of counts was computed from the entire set of identified DNA motif candidates as indicated by Sjolander et al. [65], and the parameter vector 𝛼 = (𝛼_1_, 𝛼_2,_ 𝛼_3_, 𝛼_*4*_) from a Dirichlet distribution was estimated applying function *estimateDirichDist* from the R package *usefr* available at GitHub: https://github.com/genomaths/usefr (version 0.1). As shown by Sjolander et al. [65], the posterior probabilities ${\hat{p}}_i$ are given by the expression:

4 $${\hat{p}}_i=\frac{n_i+{\alpha}_i}{\left|\overrightarrow{n}\right|+\left|\overrightarrow{\alpha}\right|}$$

where $\left|\overrightarrow{n}\right|$ and $\left|\overrightarrow{\alpha}\right|$ are the total number of counts and pseudo-counts (sum of *α*_*i*_), respectively. Random DNA MSA sequences were generated according to the estimated Dirichlet distribution with function *rdirichlet* from the R package *usefr*.

For MSAs of fixed length *N*, the *log*_2_*N* is a constant. Thus, for the purposes of MCT, we could consider the score statistic given in Eq. 2 and evaluate how much an observed aligned motif differs statistically from Monte Carlo simulated aligned sequences. The Monte Carlo *p*-value was estimated as:

5 $${p}_{\mathrm{value}}=\frac{1}{N_S+1}\sum_{i=0}^{N_s}{\delta}_i\left({S}_i,{S}_0\right)$$

where *S*_0_ is the motif score from the observed MSA to be tested, *S*_*i*_ is the motif score for the *i*th Monte Carlo simulated MSA (including *S*_0_) and $${\delta}_i\left({S}_i,{S}_0\right)=\left\{\begin{array}{c}1\ if\ {S}_i\ge {S}_0\\ {}0\ \mathrm{otherwise}\end{array}\right.$$ (*i* = 0, 1, 2, …).

Preserved methylation motifs were identified (visualized) with function *seqLogo* from Bioconductor R package *seqLogo* (version 4.1). The final visualization of DNA methylation sequence motifs was accomplished with function *ggseqlogo* from the CRAN R package *ggseqlogo* (version 0.1) [66]. DNA sequence motifs (Additional file 8) were selected based on their motif scores, all of which differed statistically from random MSA.

## Supplementary Information

Additional file 1: Figure S1. Phenotype of four selected F3 epi-lines and wild type used for methylome sequencing. Photo was taken at 27 days after planting. Epi24 and Epi8 are sister lines derived from the same crossing event; Epi10 and Epi19 are sister lines derived from a second crossing event. Figure S2. Relative DMP frequencies among different Arabidopsis epi-lines. DMPs were assigned to genic (blue), TE-related (red) and other genomic regions (green). The centroid of the wild type samples was used as reference. Relative DMP frequencies in each genomic feature were estimated as number of DMPs divided by number of total genomic cytosine positions in each genomic feature. A DMP was defined as ‘hyper’ if cytosine methylation level difference was greater than 0 and defined as ‘hypo’ if methylation level difference was less than 0. Figure S3. Total hyper- and hypo-DMP counts in epi-lines. Arabidopsis epi-lines vs WT. Each bar graph represents a single plant in each population. Cytosine context (CG, CHG, CHH) is shown separately for each plant. A DMP was defined as ‘hyper’ if cytosine methylation level difference was greater than 0 and defined as ‘hypo’ if methylation level difference was less than 0. Control centroid was used a reference. Figure S4. Upset plot showing the intersection of DMGs between different *msh1* states. The horizontal bar graph on the right (set size) shows the total number of DMGs in each dataset. The upper bar graph (intersection size) shows the number of DMGs shared by different datasets represented by black connected dots. Package *ComplexHeatmap* (version 2.0.0) in R was used to make graphs. Red arrows indicate relationship among the four epi-lines (state 4) and between selected Epi 24 (state 4) and other individual datasets representing states l-3 in our study. Figure S5. Significant enriched GO pathways by DEG analysis in Epi 8 and Epi 24. a, Enriched GO pathways shared by epi-lines Epi 8 and Epi 24 root RNA (DEG) datasets and Epi 8 leaf sample DEG dataset in Arabidopsis. Heat map was generated using the fold enrichment of GO terms (FDR < 0.05) common between the datasets. GO terms with EASE score (a modified Fisher Exact P-value) < 0.05 were used for FDR calculation. FDR was calculated using package stats (version 3.6.0; p.adjust method = FDR) in R. Package ggplot2 (version 3.3.3) in R was used to generate heatmap. b, Upset plot showing the intersection of DEGs between different Epi 8 and Epi 24 tissues. The horizontal bar graph on the right (set size) shows the total number of DEGs in each dataset. The upper bar graph (intersection size) shows the number of DEGs shared by Epi 8 and Epi 24 datasets represented by black connected dots. c, Upset plots showing the intersection of DMG and DEG datasets in Epi 8 or Epi 24. The horizontal bar graphs on the right (set size) shows the total number of DMGs or DEGs in each dataset. The upper bar graphs (intersection size) shows the number of DMGs or DEGs shared by Epi 8 datasets or Epi 24 datasets represented by black connected dots. Package *ComplexHeatmap* (version 2.0.0) in R was used to make graphs. Figure S6. Results of weighted correlation network analysis. Network derived from the 871 core *msh1*-associated DMGs resulted in 166 DMGs with Spearman’s correlation coefficient of 0.6. Loci shown in red are 33 DMGs that overlap (49%) with the 67 core hub loci from k-means cluster analysis (p-value = 0.000333 Fisher’s Exact Test). The node and label sizes are in correspondence with the node degree (from 40 to 105). That is, a large node indicates a significant correlation with a large number of other genes for its/their epigenetic effect on phenotype. Node color represents the gene score (PC-score), from light-green (small value/contribution) to dark maroon (large value/contribution). The greater the PC-score value, the greater the fraction of cumulative variance from the whole sample variance is carried by the gene and, consequently, the greater the discriminatory power carried by the gene. The pairwise correlation between genes was computed as described in Methods.

Additional file 2. Epi-line state 4 DMGs and GO enrichment.

Additional file 3. Arabidopsis C24 and Ler F1 DMGs and network enrichments.

Additional file 4. Arabidopsis *msh1* epi-line state 4 DEG and GO enrichment.

Additional file 5. 871 DMGs shared between four *msh1* states.

Additional file 6. TE and sRNA associations with 871 DMGs shared between four *msh1* states.

Additional file 7. Cluster 11 and 18 with *dcl2/3/*4-sensitive DMPs.

Additional file 8. Cluster motifs within 67 core hub loci.

Additional file 9. Review history.

## Data Availability

The datasets generated and analyzed during the current study are available from the corresponding author upon request. All next-generation sequencing data generated by this study, related to msh1 state 4, were deposited to Gene Expression Omnibus database under the following accessions: GSE192428 (Arabidopsis epi BSseq) [67], GSE192432 (Arabidopsis *msh1* memory reversion BSseq) [68], and GSE192429 (Arabidopsis epi RNAseq) [69]. Sequencing data for *msh1* states 1 and 3 can be accessed at GSE152570 [19] and data for *msh1* state 2 can be accessed under GSE129303 [18]. The customized code used in this study, including methylation analysis with Methyl-IT, DMP classification based on signal detection, and identification of DMGs, is deposited to Zenodo [70]. Codes for principal component and linear discriminant analyses are deposited to Zenodo [71].
