# Supplementary material for: Methylome decoding of RdDM-mediated reprogramming effects in the Arabidopsis MSH1 system
Source: Genome Biol. 2022 Aug 4;23:167. doi: 10.1186/s13059-022-02731-w (PMC9351182; doi:10.1186/s13059-022-02731-w)
Supplement: Supplementary file 1 — Additional file 1: Figure S1. Phenotype of four selected F3 epi-lines and wild type used for methylome sequencing. Photo was taken at 27 days after planting. Epi24 and Epi8 are sister lines derived from the same crossing event; Epi10 and Epi19 are sister lines derived from a second crossing event. Figure S2. Relative DMP frequencies among different Arabidopsis epi-lines. DMPs were assigned to genic (blue), TE-related (red) and other genomic regions (green). The centroid of the wild type samples was used as reference. Relative DMP frequencies in each genomic feature were estimated as number of DMPs divided by number of total genomic cytosine positions in each genomic feature. A DMP was defined as ‘hyper’ if cytosine methylation level difference was greater than 0 and defined as ‘hypo’ if methylation level difference was less than 0. Figure S3. Total hyper- and hypo-DMP counts in epi-lines. Arabidopsis epi-lines vs WT. Each bar graph represents a single plant in each population. Cytosine context (CG, CHG, CHH) is shown separately for each plant. A DMP was defined as ‘hyper’ if cytosine methylation level difference was greater than 0 and defined as ‘hypo’ if methylation level difference was less than 0. Control centroid was used a reference. Figure S4. Upset plot showing the intersection of DMGs between different msh1 states. The horizontal bar graph on the right (set size) shows the total number of DMGs in each dataset. The upper bar graph (intersection size) shows the number of DMGs shared by different datasets represented by black connected dots. Package ComplexHeatmap (version 2.0.0) in R was used to make graphs. Red arrows indicate relationship among the four epi-lines (state 4) and between selected Epi 24 (state 4) and other individual datasets representing states l-3 in our study. Figure S5. Significant enriched GO pathways by DEG analysis in Epi 8 and Epi 24. a, Enriched GO pathways shared by epi-lines Epi 8 and Epi 24 root RNA (DEG) datasets and Epi 8 leaf sam [file 13059_2022_2731_MOESM1_ESM.pptx]

## Slide 1
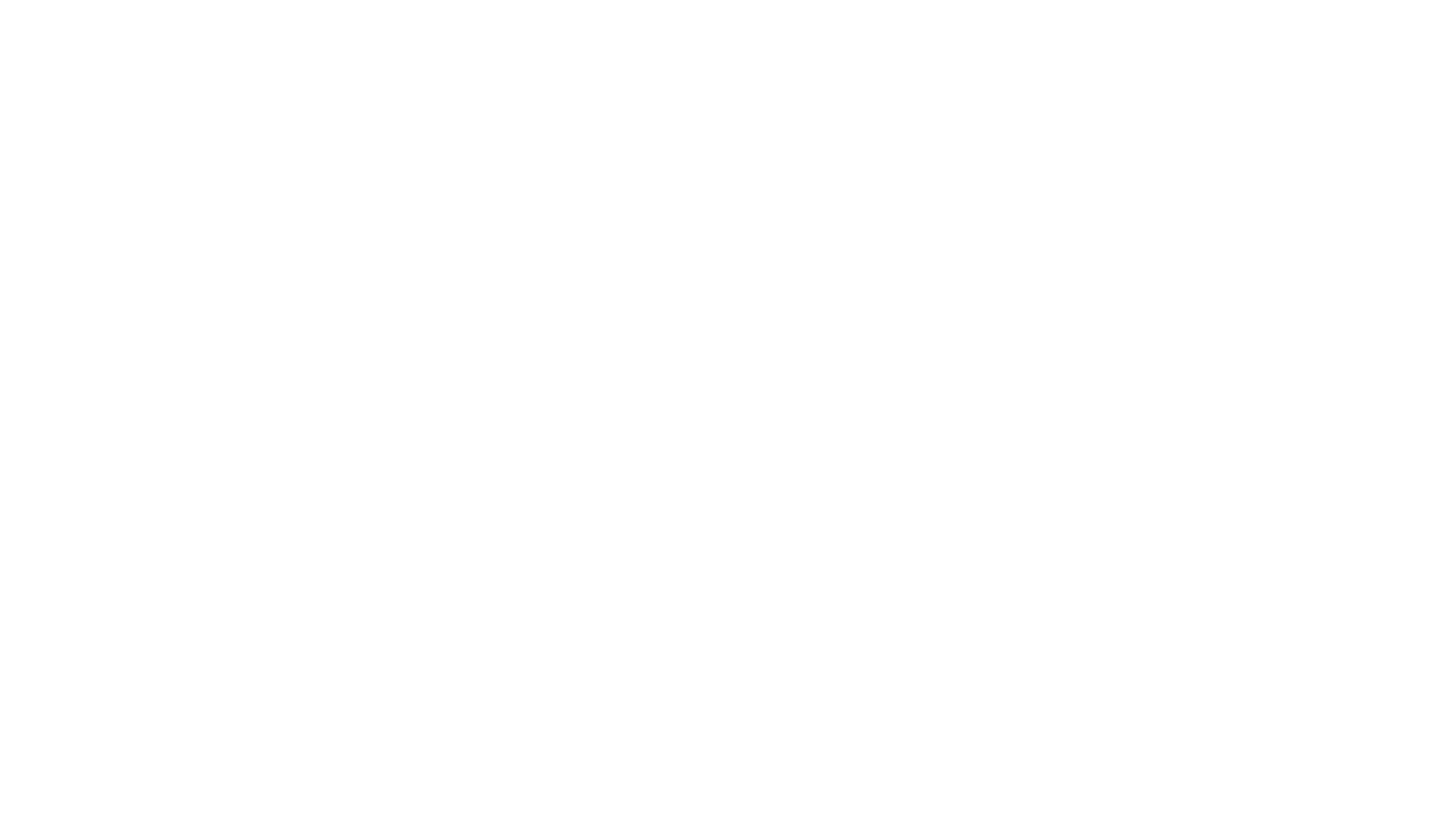

#

## Slide 2
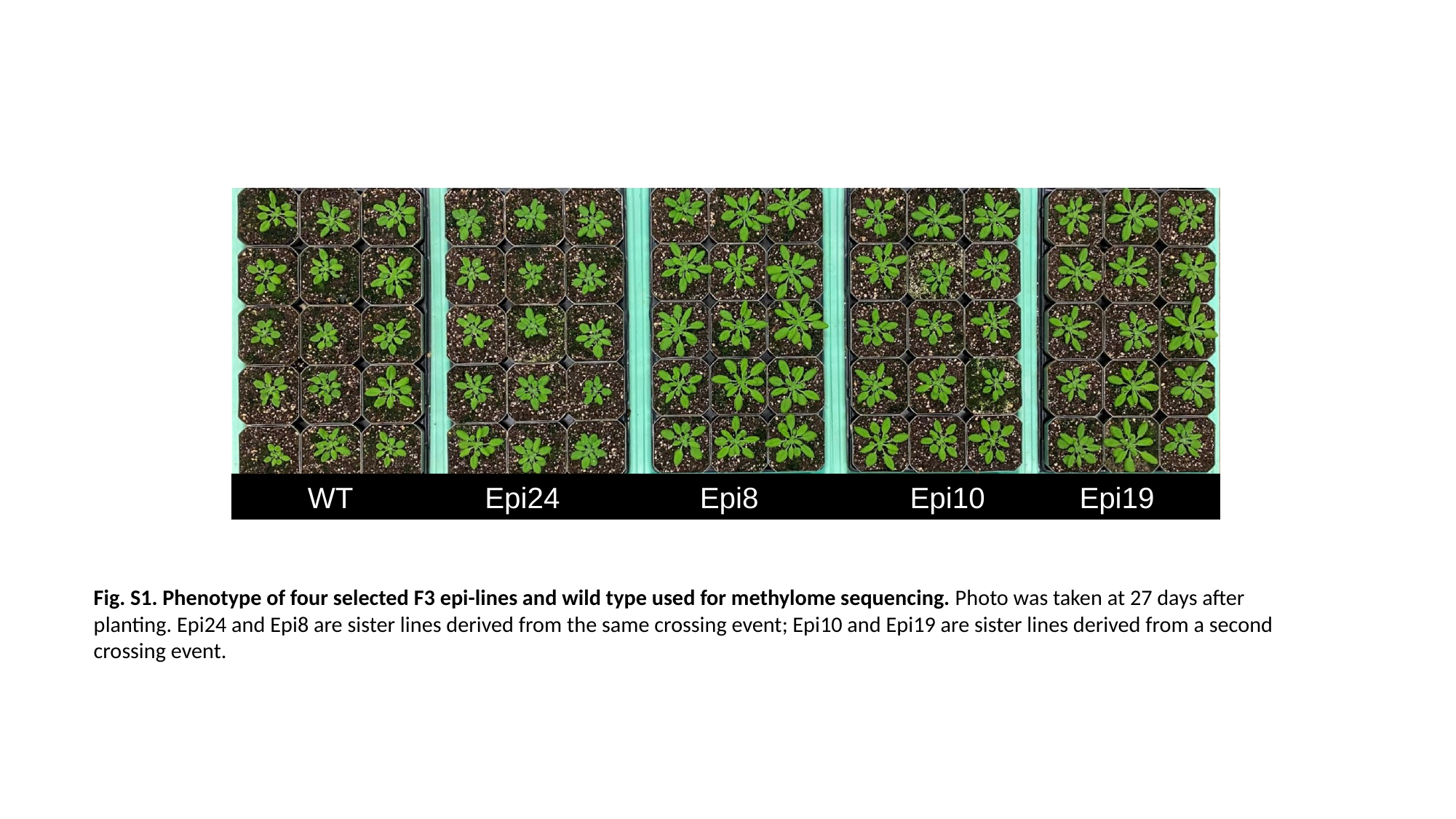

WT 	 Epi24	 Epi8	 Epi10	 Epi19
Fig. S1. Phenotype of four selected F3 epi-lines and wild type used for methylome sequencing. Photo was taken at 27 days after planting. Epi24 and Epi8 are sister lines derived from the same crossing event; Epi10 and Epi19 are sister lines derived from a second crossing event.

## Slide 3
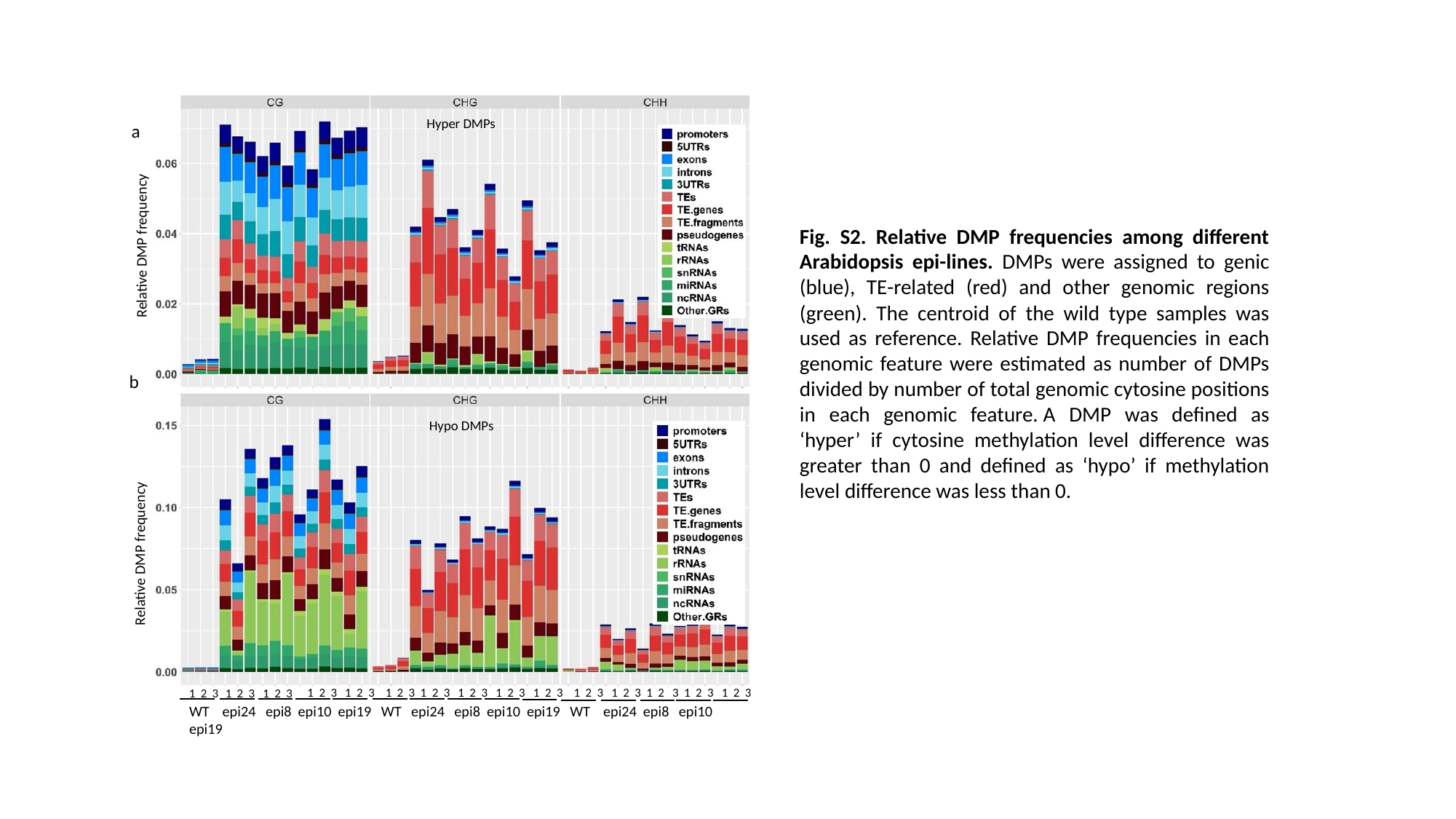

Hyper DMPs
Relative DMP frequency
Hypo DMPs
Relative DMP frequency
1 2 3 1 2 3 1 2 3 1 2 3 1 2 3 1 2 3 1 2 3 1 2 3 1 2 3 1 2 3 1 2 3 1 2 3
 1 2 3
1 2 3
1 2 3
WT epi24 epi8 epi10 epi19 WT epi24 epi8 epi10 epi19 WT epi24 epi8 epi10 epi19
a
b
Fig. S2. Relative DMP frequencies among different Arabidopsis epi-lines. DMPs were assigned to genic (blue), TE-related (red) and other genomic regions (green). The centroid of the wild type samples was used as reference. Relative DMP frequencies in each genomic feature were estimated as number of DMPs divided by number of total genomic cytosine positions in each genomic feature. A DMP was defined as ‘hyper’ if cytosine methylation level difference was greater than 0 and defined as ‘hypo’ if methylation level difference was less than 0.

## Slide 4
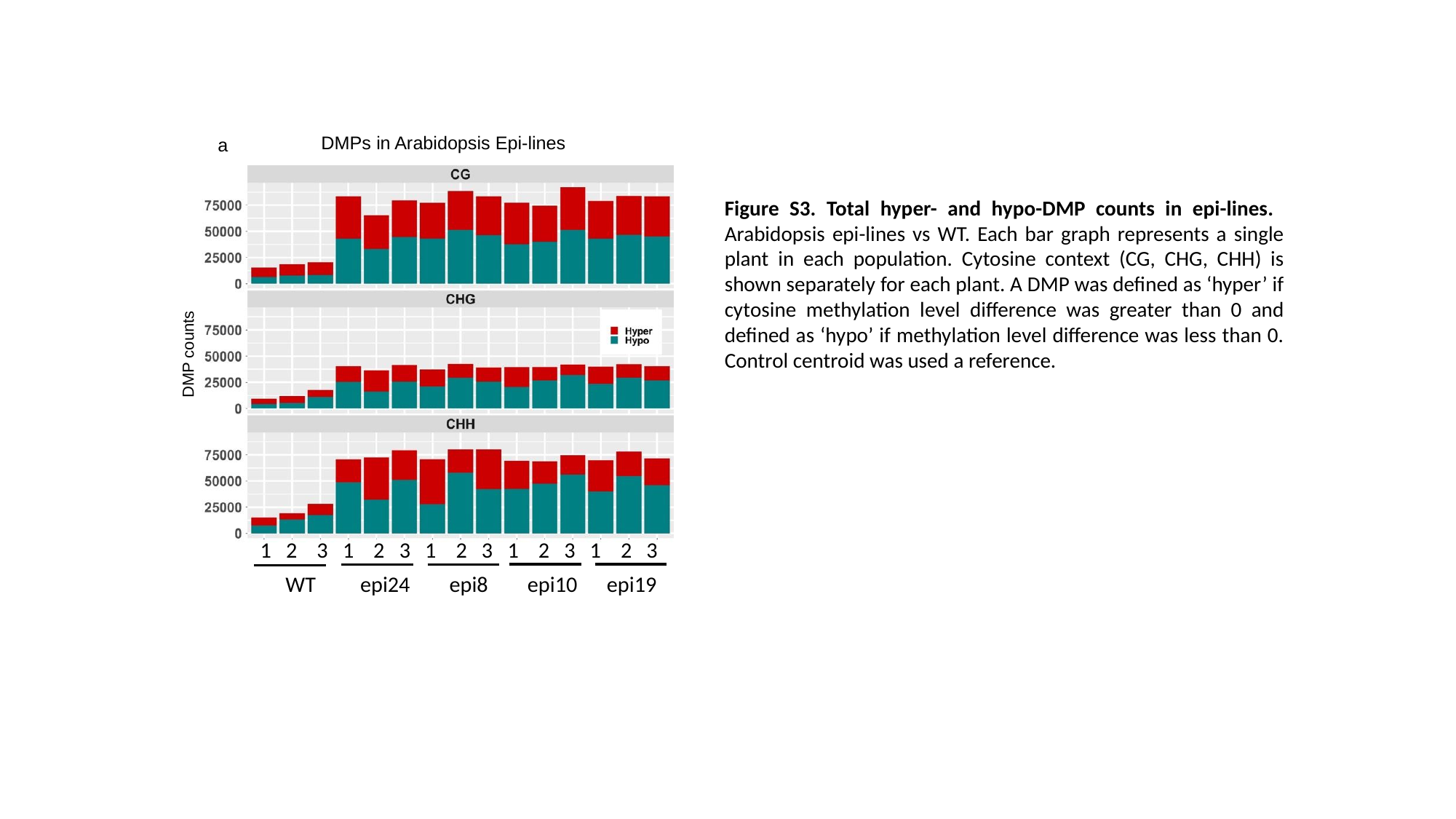

DMPs in Arabidopsis Epi-lines
a
DMP counts
 1 2 3 1 2 3 1 2 3 1 2 3 1 2 3
WT epi24 epi8 epi10 epi19
Figure S3. Total hyper- and hypo-DMP counts in epi-lines. Arabidopsis epi-lines vs WT. Each bar graph represents a single plant in each population. Cytosine context (CG, CHG, CHH) is shown separately for each plant. A DMP was defined as ‘hyper’ if cytosine methylation level difference was greater than 0 and defined as ‘hypo’ if methylation level difference was less than 0. Control centroid was used a reference.

## Slide 5
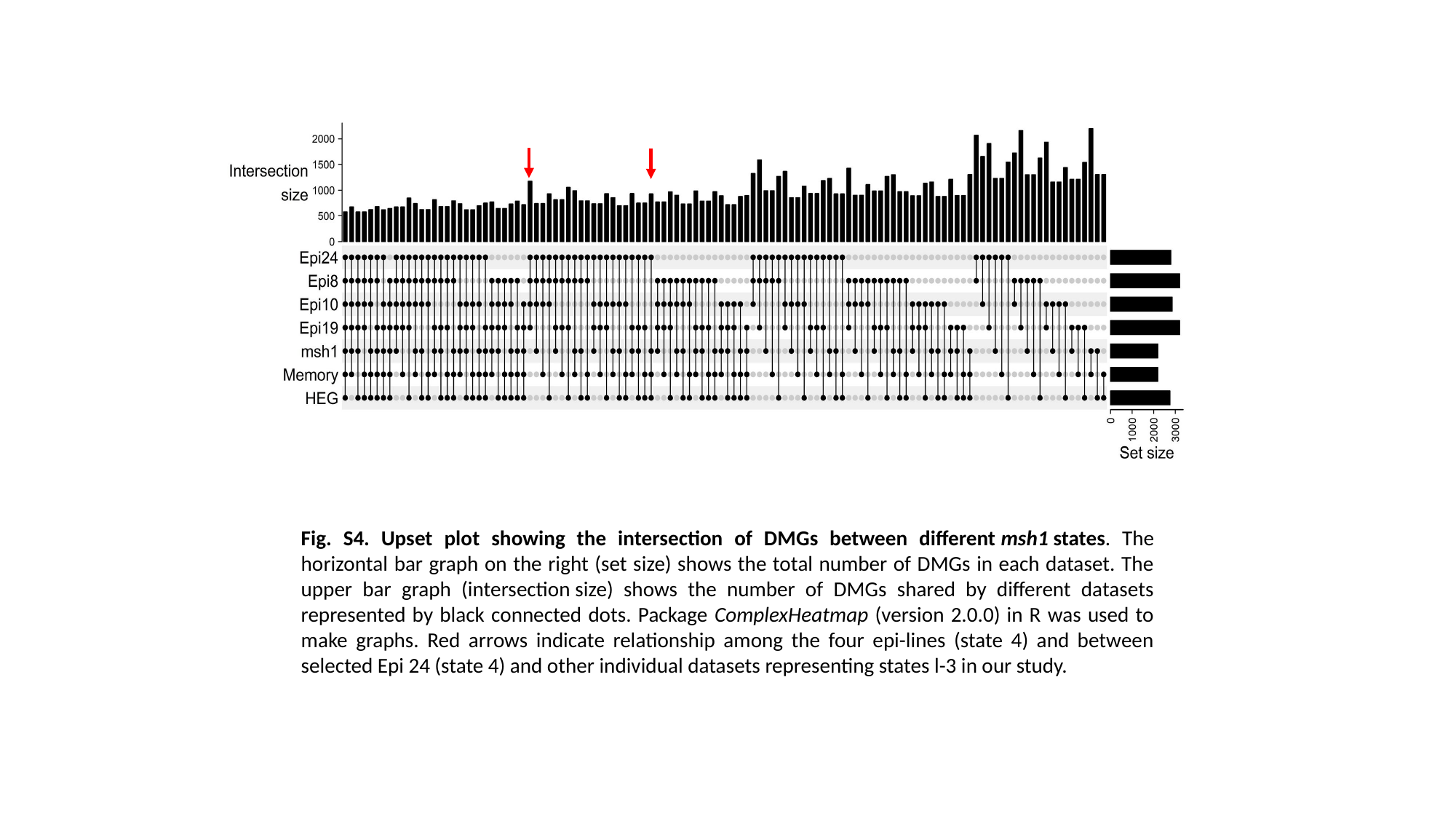

Fig. S4. Upset plot showing the intersection of DMGs between different msh1 states. The horizontal bar graph on the right (set size) shows the total number of DMGs in each dataset. The upper bar graph (intersection size) shows the number of DMGs shared by different datasets represented by black connected dots. Package ComplexHeatmap (version 2.0.0) in R was used to make graphs. Red arrows indicate relationship among the four epi-lines (state 4) and between selected Epi 24 (state 4) and other individual datasets representing states l-3 in our study.

## Slide 6
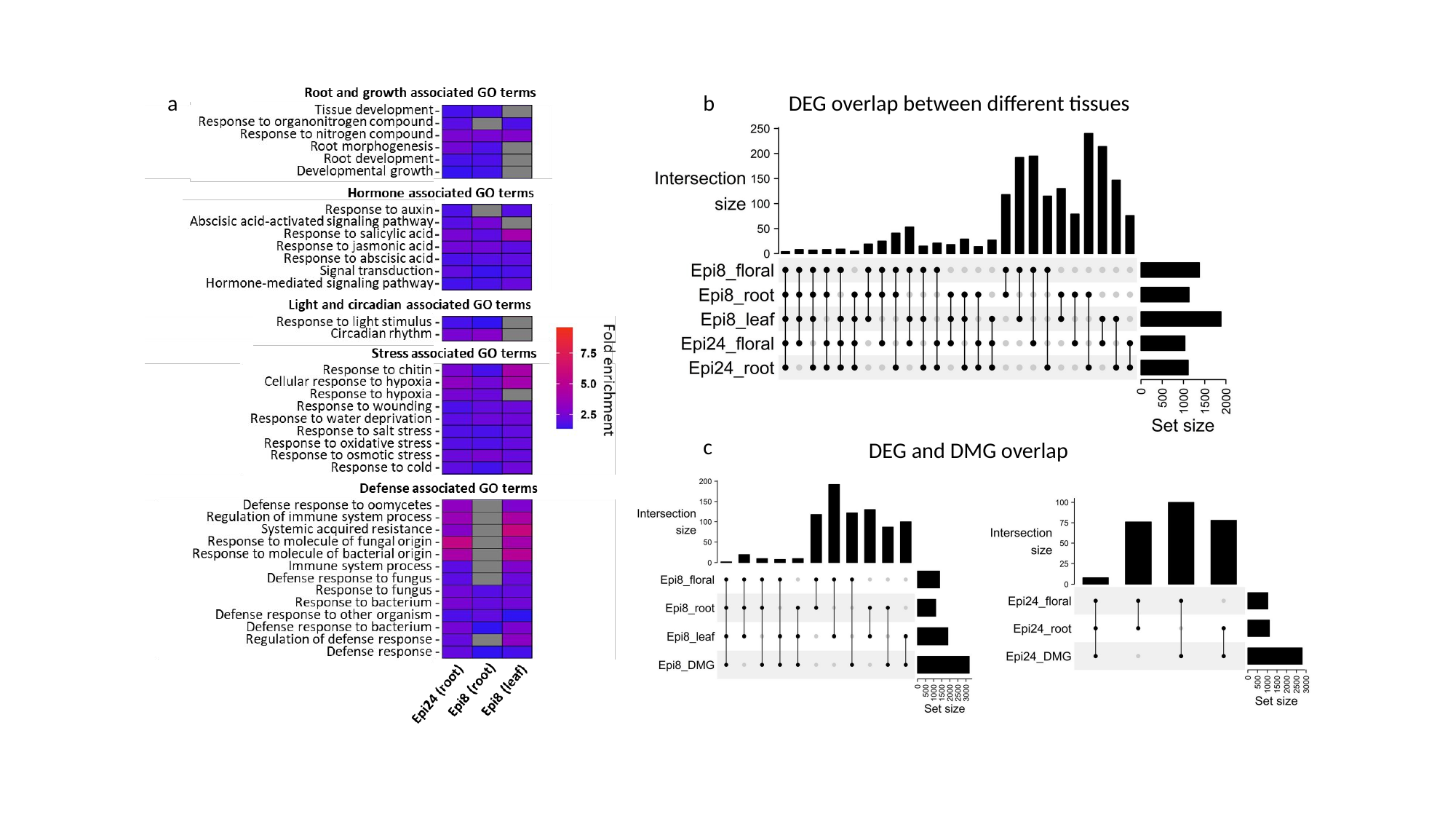

a
b
DEG overlap between different tissues
c
DEG and DMG overlap

## Slide 7
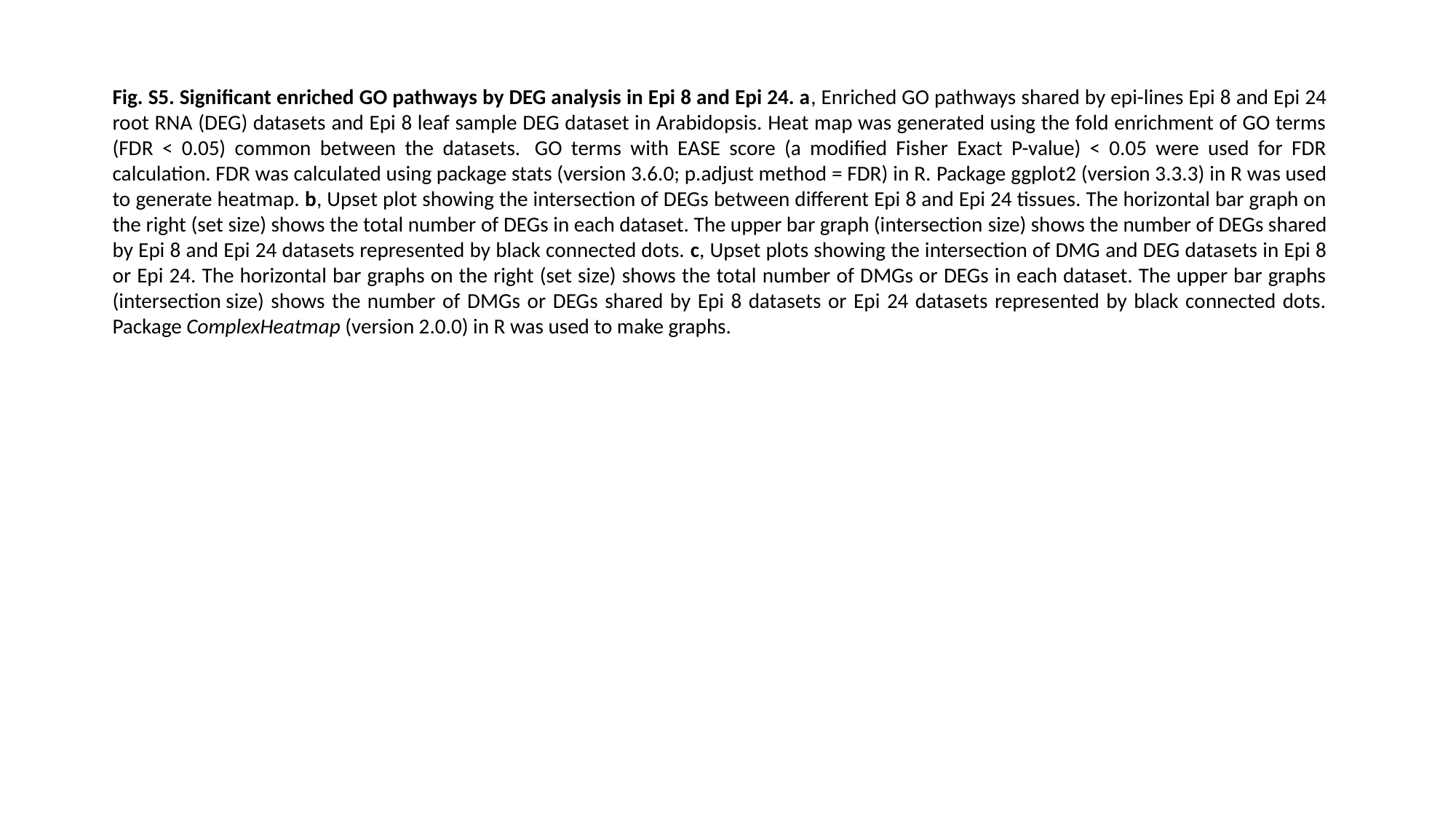

Fig. S5. Significant enriched GO pathways by DEG analysis in Epi 8 and Epi 24. a, Enriched GO pathways shared by epi-lines Epi 8 and Epi 24 root RNA (DEG) datasets and Epi 8 leaf sample DEG dataset in Arabidopsis. Heat map was generated using the fold enrichment of GO terms (FDR < 0.05) common between the datasets.  GO terms with EASE score (a modified Fisher Exact P-value) < 0.05 were used for FDR calculation. FDR was calculated using package stats (version 3.6.0; p.adjust method = FDR) in R. Package ggplot2 (version 3.3.3) in R was used to generate heatmap. b, Upset plot showing the intersection of DEGs between different Epi 8 and Epi 24 tissues. The horizontal bar graph on the right (set size) shows the total number of DEGs in each dataset. The upper bar graph (intersection size) shows the number of DEGs shared by Epi 8 and Epi 24 datasets represented by black connected dots. c, Upset plots showing the intersection of DMG and DEG datasets in Epi 8 or Epi 24. The horizontal bar graphs on the right (set size) shows the total number of DMGs or DEGs in each dataset. The upper bar graphs (intersection size) shows the number of DMGs or DEGs shared by Epi 8 datasets or Epi 24 datasets represented by black connected dots. Package ComplexHeatmap (version 2.0.0) in R was used to make graphs.

## Slide 8
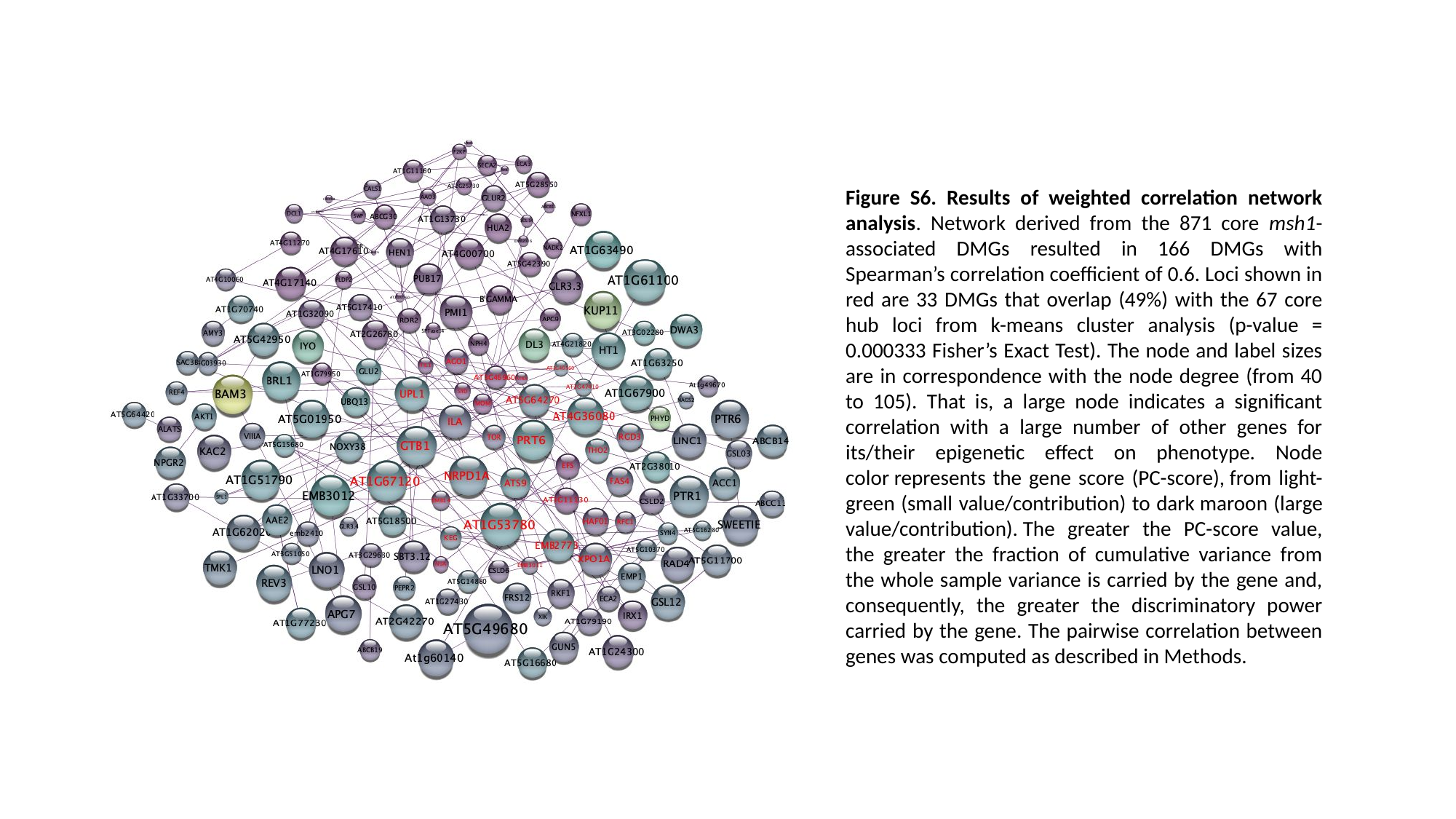

Figure S6. Results of weighted correlation network analysis. Network derived from the 871 core msh1-associated DMGs resulted in 166 DMGs with Spearman’s correlation coefficient of 0.6. Loci shown in red are 33 DMGs that overlap (49%) with the 67 core hub loci from k-means cluster analysis (p-value = 0.000333 Fisher’s Exact Test). The node and label sizes are in correspondence with the node degree (from 40 to 105). That is, a large node indicates a significant correlation with a large number of other genes for its/their epigenetic effect on phenotype. Node color represents the gene score (PC-score), from light-green (small value/contribution) to dark maroon (large value/contribution). The greater the PC-score value, the greater the fraction of cumulative variance from the whole sample variance is carried by the gene and, consequently, the greater the discriminatory power carried by the gene. The pairwise correlation between genes was computed as described in Methods.
