# Supplementary material for: Methylome decoding of RdDM-mediated reprogramming effects in the Arabidopsis MSH1 system
Source: Genome Biol. 2022 Aug 4;23:167. doi: 10.1186/s13059-022-02731-w (PMC9351182; doi:10.1186/s13059-022-02731-w)
Supplement: Supplementary file 9 — Additional file 9. Review history. [file 13059_2022_2731_MOESM9_ESM.docx]

Review History

**First round of review**

**Reviewer 1**

**Were you able to assess all statistics in the manuscript, including the appropriateness of statistical tests used?**

No, I do not feel adequately qualified to assess the statistics.

**Comments to author:**

This is a very interesting paper on determining the role of RdDM-dependent methylation changes within identifiable sequence motifs to determine gene hubs within networks that discriminate stress response and growth vigor epigenetic phenotypes in Arabidopsis. The experimental strategy is well-designed and quite informative. I enjoyed reading this manuscript and have a few points that the authors need to address before their paper is accepted for publication.

-Changes in gene expression between different epi-lines and the WT was one by analyzing floral stem tissues because MSH1 expression is enriched in this tissue. However, the most notable phenotypic changes were observed in shoot and root area and biomass accumulation. The epigenetic effects, as expected, are observed in tissues where the causal gene is not expressed if the alterations are inheritable. It is necessary to analyze changes in gene expression in root and/or the shoot to confirm that the epigenetic changes do not require MSH1 activity.

- Related to the above comment, the authors analyze gene expression in floral stems and identify PPI networks specific for the two epigenetic populations examined EPI 8 and Epi 24. Although the data is extracted from floral stems, they extrapolate the effect of these PPI networks to all vegetative tissue, such an effect on ribosome biogenesis-related gene expression that is used to explain the enhanced vegetative growth in Epi8. The authors also identify response to response to auxin as a pathway affected in both Epi lines; since auxin plays a major role in root development, including primary root growth and lateral root formation, it would be very interesting to test how the auxin response pathway is affected in the roots of these Epi lines, as it promotes root development without having negative effect on the root or shoot development, suggesting that changes in gene expression are probably reflected at the tissue and/or cell line level. This analysis is also important because several DMG categories such as root development are not reflected in the DEG analysis.

The authors mention in the discussion section that "DNA methylation and gene expression analysis in this study provided unambiguous discrimination of the four msh1-derived states on a uniform Col-0 genetic background. Yet, it was possible to identify a subset of 871 DMGs shared among the four distinct states. We interpret this finding to indicate that reprogramming events in the original msh1 mutant are foundational to the other derived states." It would be very interesting to have a more in-depth discussion as to how the State1 and State 2 epigenetic reprograming, that is reflected in an apparent lost in fitness, is "reprogrammed" by crossing WT or grafting to a phenotype of apparent gain of fitness. This involved also, perhaps speculation, of how a stable core of PPI nodes are established to produce a beneficial phenotype.

**Reviewer 2**

**Were you able to assess all statistics in the manuscript, including the appropriateness of statistical tests used?**

No, I do not feel adequately qualified to assess the statistics.

**Comments to author:**

This is an interesting report on msh1 mutant, attempting to explain diversity of various msh1-triggered phenotypes. The authors performed partial phenotypic analysis, methylome analysis and transcriptome analysis. The authors claim that they identified clusters/pathways specifically targeted by RdDM machinery.

The main gaps in the manuscript are:

- lack of definitive analysis of correlation between methylome and transcriptome;

- lack of ncRNA-seq - thus speculation about inheritance of certain ncRNA pools are not justified

- lack of integrative analysis between ncRNA-seq, methylome and transcriptome to justify that differential expression of ncRNA triggers specific methylome changes that lead to specific transcriptome changes that change the phenotype

On occasion, we observed sporadic, low frequency incidence of reversion to a condition resembling memory (state 2) phenotype (Fig. 3a) - to me, this is not a low frequency, as per the image you presented

". The procedure incorporates signal detection and machine learning" - was there actually a machine learning implemented?

"DNA methylation variation within wild type samples was measured and subtracted" - subtracted from what?

No correlation between DMG and DEG was done

No ncRNA-seq was done - target prediction was mostly based on methylation?

"…suggest that transgenerational stability involves re-establishment of de novo methylation sites by inherited sRNAs each generation" - so, this should have been done - comparison of sRNA pools.

"…that interaction of the msh1 memory genome with naïve 459 (WT) through reciprocal…" - naïve genome?

It would be interesting to compare methylome/transcriptome of revertants and the epimutants.

The authors used "females" for crosses, to "deliver" epigenetic factors through cytoplasm - it would be good to provide the indication that transgenerational inheritance is indeed predominantly based on cytoplasmic inheritance (like for example the evidence that the ncRNA pool in the nucleus is negligible).

The authors claim they used machine learning approaches - I did not find any evidence of that.

**Authors Response**

**Point-by-point responses to the reviewers’ comments:**

Reviewer #1:

This is a very interesting paper on determining the role of RdDM-dependent methylation changes within identifiable sequence motifs to determine gene hubs within networks that discriminate stress response and growth vigor epigenetic phenotypes in Arabidopsis. The experimental strategy is well-designed and quite informative. I enjoyed reading this manuscript and have a few points that the authors need to address before their paper is accepted for publication.

-Changes in gene expression between different epi-lines and the WT was one by analyzing floral stem tissues because MSH1 expression is enriched in this tissue. However, the most notable phe-notypic changes were observed in shoot and root area and biomass accumulation. The epigenetic effects, as expected, are observed in tissues where the causal gene is not expressed if the altera-tions are inheritable. It is necessary to analyze changes in gene expression in root and/or the shoot to confirm that the epigenetic changes do not require MSH1 activity.

*Response: We appreciate and agree with this comment. We now include an additional supplemen-tary figure that describes changes in root transcriptome data between two selected epi-lines that differ in phenotype (Epi 24 and Epi 8), and between root and shoot tissues from a single epi-line (Epi-8). This addition shows that some differences exist between tissues and that floral tissues pro-vide the most meaningful resolution of the msh1 effect.*

- Related to the above comment, the authors analyze gene expression in floral stems and identify PPI networks specific for the two epigenetic populations examined EPI 8 and Epi 24. Although the data is extracted from floral stems, they extrapolate the effect of these PPI networks to all vegeta-tive tissue, such an effect on ribosome biogenesis-related gene expression that is used to explain the enhanced vegetative growth in Epi8. The authors also identify response to response to auxin as a pathway affected in both Epi lines; since auxin plays a major role in root development, including primary root growth and lateral root formation, it would be very interesting to test how the auxin response pathway is affected in the roots of these Epi lines, as it promotes root development with-out having negative effect on the root or shoot development, suggesting that changes in gene ex-pression are probably reflected at the tissue and/or cell line level. This analysis is also important because several DMG categories such as root development are not reflected in the DEG analysis.

*Response: With addition of the new supplementary figure, we show that Epi8 and Epi24 root da-tasets differ in both response to auxin and defense response pathways.*

The authors mention in the discussion section that "DNA methylation and gene expression analysis in this study provided unambiguous discrimination of the four msh1-derived states on a uniform Col-0 genetic background. Yet, it was possible to identify a subset of 871 DMGs shared among the four distinct states. We interpret this finding to indicate that reprogramming events in the original msh1 mutant are foundational to the other derived states." It would be very interesting to have a more in-depth discussion as to how the State1 and State 2 epigenetic reprograming, that is reflect-ed in an apparent lost in fitness, is "reprogrammed" by crossing WT or grafting to a phenotype of apparent gain of fitness. This involved also, perhaps speculation, of how a stable core of PPI nodes are established to produce a beneficial phenotype

*Response: We agree that a vital question in the msh1 system is the dichotomy of phenotype arising in msh1 and memory for heightened/sustained stress response and in graft or crossed progeny for heightened vigor. We have addressed this in the Discussion by pointing to work by others that links several of the genes identified in the 67 putative network hubs as being central phenotype “pivot points” for growth decisions in the plant. These include TOR, SYD and BRM, which are central inte-grators of stress versus growth. We don’t consider it coincidental that these genes appear linked to organellar properties via MSH1, since mitochondria and plastids serve both as environmental sen-sors and energy modulators of the cell. But to speculate further at this point is difficult within the context of the current study. We are also being mindful to avoid overinterpreting our data at this point. As suggested by the reviewer, we are working to address the stability of these core-hubs through multi-generational state 2 memory material.*

Reviewer #2:

This is an interesting report on msh1 mutant, attempting to explain diversity of various msh1-triggered phenotypes. The authors performed partial phenotypic analysis, methylome analysis and transcriptome analysis. The authors claim that they identified clusters/pathways specifically target-ed by RdDM machinery.

The main gaps in the manuscript are:

- lack of definitive analysis of correlation between methylome and transcriptome;

- lack of ncRNA-seq - thus speculation about inheritance of certain ncRNA pools are not justified

- lack of integrative analysis between ncRNA-seq, methylome and transcriptome to justify that dif-ferential expression of ncRNA triggers specific methylome changes that lead to specific transcrip-tome changes that change the phenotype

*Response: We have addressed these concerns within the text below as they are raised.*

On occasion, we observed sporadic, low frequency incidence of reversion to a condition resem-bling memory (state 2) phenotype (Fig. 3a) - to me, this is not a low frequency, as per the image you presented

*Response: The image shows just one pot, that with the highest number of revertant plants of mul-tiple pots. We have shown one sample epi-line planting, but often obtain plantings with no rever-sion. We attempt to be comprehensive in our report, as shown in the numbers reported in Table 1, while relying on a figure that is artificially higher frequency for visual emphasis.*

". The procedure incorporates signal detection and machine learning" - was there actually a ma-chine learning implemented?

*Response: The methylome analysis platform that we implement in the study, Methyl-IT, relies on signal detection and machine learning to allow us to identify those DMPs that have the highest probability of treatment (msh1) association. The classification performance of the machine learn-ing model classifier is evaluated in a bootstrap analysis including about 18 classification perfor-mance indicators such as, for example, accuracy, accuracy 95% confidence interval, Balanced Accu-racy, Sensitivity, Specificity, Detection Rate, FDR, etc. This methodology, published and demon-strated previously, is described in the citations provided and several tutorials are available at: https://genomaths.github.io/methylit/index.html.*

"DNA methylation variation within wild type samples was measured and subtracted" - subtracted from what?

*Response: DNA methylation variation is detected among wild type samples, arising due to natural chromatin fluctuations, during developmental transitions or in response to environmental varia-tion. This variation is detected by comparing individual wild type samples with a centroid of wild type. We consider this WT variation as ‘background’ for our study, and we subtract this from varia-tion detected in msh1-derived (treatment) samples by estimating the information divergence be-tween each sample group and the centroid. The information divergences currently in use are: Hellinger divergence, total variation distance (absolute value of methylation levels difference), and J-divergence (symmetric form of the relative entropy, also known as Kullback-Leibler divergence). The further application of signal detection and machine learning involves discrimination (enrich-ment for) methylation variation with highest probability of treatment association. By this means, wild type variation is measured and subtracted from total variation, and the methodology is de-scribed in detail in the reports cited (25-Sanchez and Mackenzie, 36-Sanchez et al.).*

No correlation between DMG and DEG was done.

No ncRNA-seq was done - target prediction was mostly based on methylation?

*Response: We are not sure that we understand what the reviewer is seeking, but we wish to ad-dress two aspects of these concerns. As mentioned above, we have now added supplementary data to further elaborate transcriptome features of the epi-lines. However, with regard to DMG and DEG “correlation”, we make clear in the Discussion that no such direct correlation exists either in our datasets or in those reported by others. The reasoning for this likely goes beyond the scope of this report.*

*We explicitly incorporate novel methylome analysis methods in our study to investigate intragenic, low-density methylation variation. As we show, analysis of this variation allows the identification of RdDM targets, functionally verified by sRNA cluster and dcl2/3/4 mutant analysis. However, we do not present physical evidence for how this intragenic, RdDM-directed methylation variation im-pacts gene expres sion or phenotype. Our group has separately developed evidence that the RdDM target loci can undergo changes in alternative splicing in association with methylation repatterning. Long-read (Nanopore) RNAseq analysis of leaf, root and reproductive tissues permits us to evaluate alternative splicing behavior in wild type versus msh1-derived states. We focus these studies cur-rently on the identified 67 putative hub genes that undergo state-specific differential methyla-tion, with evidence of methylome influences undetectable by standard RNAseq data. In the table shown below, for example, we summarize a portion of this information. We present 17 of the 67 (25%) putative hub loci that display differential isoform accumulation in Epi 10 versus wild type leaf RNA samples. The study of alternative splicing involves analysis of multiple datasets from 4 msh1 states x 3 developmental stages for comprehensive documentation of methylome repattern-ing-RNA splicing association. These data extend well beyond what could practically be incorporated here. The data we present in our current report are vital to understanding the relationship of DNA methylome repatterning to genic RNA behavior. We intend to report the study of alternative splic-ing behavior in a separate, subsequent publication.*

*(please see table in attached response to reviewer document uploaded)*

"…suggest that transgenerational stability involves re-establishment of de novo methylation sites by inherited sRNAs each generation" - so, this should have been done - comparison of sRNA pools.

*Response: The experiments proposed by the reviewer differ in several ways from the approach we have taken. We do not find that comparison of sRNA pools is informative in transgenerational anal-ysis without the benefit of dcl2/3/4 mutant analysis to complement our analysis. Methylome and sRNA datasets are inherently stochastic transgenerationally, so it is difficult to achieve sufficient resolution to carry out the experiments suggested.*

"…that interaction of the msh1 memory genome with naïve 459 (WT) through reciprocal…" - naïve genome?

*Response: we use the term ‘naïve’ when referring to a genome that has not been exposed to epi-genetic reprogramming. This terminology is consistent with its use in the literature more generally when referring to incorporation of epigenetic mutants.*

It would be interesting to compare methylome/transcriptome of revertants and the epimutants.

The authors used "females" for crosses, to "deliver" epigenetic factors through cytoplasm - it would be good to provide the indication that transgenerational inheritance is indeed predominantly based on cytoplasmic inheritance (like for example the evidence that the ncRNA pool in the nucle-us is negligible).

The authors claim they used machine learning approaches - I did not find any evidence of that.

*Response: we are not sure that we understand what the reviewer is seeking here, but it is im-portant to make clear that we do not use the msh1 source as female in any of our experiments, unless to demonstrate outcomes of reciprocal crossing. The msh1 materials are generally used as male because we recognize that MSH1 is an organellar (mitochondrial and plastid) protein, so its incorporation as pollen parent avoids potential confounding cytoplasmic effects.*

*We have responded to the question regarding machine learning applications through Methyl-IT.*

**Second round of review**

**Reviewer 1**

I am satisfied with the ansewers to my questions and comments. However, I could not find in the paper ebough information about the way machine learning was used for network construction. The authors used a methylome analysis platform that they implement in the study, Methyl-IT,

relies on signal detection and machine learning to allow identification of DMPs with highest probability of treatment association. The pipeline and parameters used for this study should be included in detail in this manuscript. The use of machine learning is becaoming very powerful for network analysis and it would be beneficial to other research group to have a full description of the ML strategies and pipilenes used. If the the pipeline and software ysed are available in a publicaly accesible website, it should be clearly presented in the text and material and methods section.

**Reviewer 2**

The authors responded to my questions.

**Authors Response**

**Point-by-point responses to the reviewers’ comments:**

Reviewer #1:

I am satisfied with the ansewers to my questions and comments. However, I could not find in the paper ebough information about the way machine learning was used for network construction. The authors used a methylome analysis platform that they implement in the study, Methyl-IT,

relies on signal detection and machine learning to allow identification of DMPs with highest probability of treatment association. The pipeline and parameters used for this study should be included in detail in this manuscript. The use of machine learning is becaoming very powerful for network analysis and it would be beneficial to other research group to have a full description of the ML strategies and pipilenes used. If the the pipeline and software ysed are available in a publicaly accesible website, it should be clearly presented in the text and material and methods section.

*Response: We have made the requested formatting changes to the manuscript. However, we wish to make one clarification. Reviewer 1 requested additional detail about the machine learning methodologies that were implemented for gene network analysis. We have made clear within the Methods section that, for the downstream network analysis, we used Cytoscape with K-means clustering, an unsupervised machine learning algorithm, which was used as implemented in Cytoscape. No additional detail should be necessary to reproduce our results.*
